# Glycosylation of bacterial antigens changes epitope patterns

**DOI:** 10.3389/fimmu.2023.1258136

**Published:** 2023-10-26

**Authors:** Karolin Kern, Nicolas Delaroque, Anders Boysen, Marcus Puder, Ralph Wendt, Andreas Kölsch, Eva Ehrentreich-Förster, Kristian Stærk, Thomas Emil Andersen, Karin Andersen, Lars Lund, Michael Szardenings

**Affiliations:** ^1^ Ligand Development Unit, Fraunhofer Institute for Cell Therapy and Immunology (IZI), Leipzig, Germany; ^2^ Epitopic, Leipzig, Germany; ^3^ GlyProVac ApS, Odense, Denmark; ^4^ Department of Nephrology, St. Georg Hospital Leipzig, Leipzig, Germany; ^5^ MicroDiagnostics Unit, Fraunhofer Institute for Cell Therapy and Immunology (IZI), Leipzig, Germany; ^6^ Molekulare und Zelluläre Bioanalytik Unit, Fraunhofer Institute for Cell Therapy and Immunology, Branch Bioanalytics and Bioprocesses (IZI-BB), Golm, Germany; ^7^ Department of Clinical Microbiology, Odense University Hospital, Odense, Denmark; ^8^ Research Unit of Clinical Microbiology, University of Southern Denmark, Odense, Denmark; ^9^ Department of Urology, Odense University Hospital, Odense, Denmark; ^10^ Department of Clinical Research, University of Southern Denmark, Odense, Denmark

**Keywords:** vaccine development, uropathogenic, protein glycosylation, *Escherichia coli*, SSLE, YghJ, ACFD, epitope

## Abstract

**Introduction:**

Unlike glycosylation of proteins expressed in mammalian systems, bacterial glycosylation is often neglected in the development of recombinant vaccines.

**Methods:**

Here, we compared the effects of glycosylation of YghJ, an *Escherichia coli* protein important for mucus attachment of bacteria causing in urinary tract infections (UTIs). A novel method based on statistical evaluation of phage display for the identification and comparison of epitopes and mimotopes of anti-YghJ antibodies in the sera was used. This is the first time that the effect of glycosylation of a recombinant bacterial antigen has been studied at the peptide epitope level.

**Results:**

The study identifies differences in the immune response for (non)-glycosylated antigens in rabbits and pigs and compares them to a large group of patients with UTI, which have been diagnosed as positive for various bacterial pathogens. We identified glycosylation-specific peptide epitopes, a large immunological similarity between different UTI pathogens, and a broad peptide epitope pattern in patients and animals, which could result in a variable response in patients upon vaccination.

**Discussion:**

This epitope analysis indicates that the vaccination of rabbits and pigs raises antibodies that translate well into the human immune system. This study underlines the importance of glycosylation in bacterial vaccines and provides detailed immune diagnostic methods to understand individual immune responses to vaccines.

## 1 Introduction

The Uropathogenic *Escherichia coli* (UPEC) is among the pathogens that exert the largest burden on public health and healthcare budgets in high-income countries (1). UPEC not only causes simple and recurrent urinary tract infections (UTIs), but also is associated with complicated UTIs. The lifetime incidence of a simple UTI is 50%–60% in adult women, and even with acute treatment, recurrence infections are confirmed within 6–12 months (2). Although recurrent UTIs are viewed as a condition with low morbidity, infections have a negative impact on the quality of life (3). Complicated UTIs are often healthcare-associated UTIs (HAUTI), where the infection is a consequence of the intervention of another disease or condition in at-risk patient groups. HAUTI risk groups include patients with diabetes, kidney stones, spinal cord dysfunction, and all surgical patients, as urinary catheters are one of the main risk factors (4, 5). The probability of developing complex HAUTIs increases dramatically with age above 60 (6). UPEC is the most prevalent causative agent of HAUTI, as it is isolated in approximately one-third of cases (26%–47%) (6–8). Despite an unmet need, there is currently no marketed vaccine targeting this pathogen.

The UTI burden is predicted to increase as UPEC develops concerning levels of resistance to extended spectrum cephalosporine, aminoglycosides, tetracyclines, and fluoroquinolones (9). This has made UPEC a CDC and WHO priority pathogen and emphasizes the need for alternative interventions to combat antimicrobial resistance (AMR) in this pathogen (9–11). Among the proposed strategies for AMR prevention, vaccines have been highlighted as an attractive mode of intervention.

The impact of UPEC on the individual patient as well as entire healthcare systems has driven the research to understand UPEC pathogenicity and to develop new approaches and vaccine technologies for preventing the disease. One new vaccine approach is attempting to exploit the discovery of extensive O-linked protein glycosylation in *E. coli* (12). O-linked protein glycosylation is the covalent linking of glycans to either a Serine or Threonine (13). Although this type of post-translational modification is common in eukaryotic cells, O-linked protein glycosylation has been reported only in a few bacterial pathogens (12–18). In an earlier study, the abundance of outer membrane-associated glycoproteins in Enterotoxigenic *E. coli* (ETEC) was described (12). It has been suggested that the controlled inclusion of O-linked glycosylations in protein-based subunit vaccines could result in a more differential immune response and, thus, higher efficacy compared to an antigen without these modifications.

The protein YghJ was selected by GlyProVac LLC as a lead antigen for glycosylation in their efforts to develop a vaccine candidate targeting UPEC HAUTI. YghJ, also known as either SslE or AcfD, is a secreted metalloprotease that degrades the protective mucus layer secreted by epithelial cells, thereby allowing the pathogen to attach to the underlying cells (19, 20). This protein displays a high degree of conservation across the entire *E. coli* family, and has been shown to be glycosylated at more than 50 sites in two different ETEC strains (21–23). One of the most important attributes of YghJ is the immunogenic property of glycans attached to the protein.

Glycosylated YghJ (gYghJ) has been investigated *in vitro* using sera from animals immunized with the antigen or sera isolated from human volunteers who had participated in controlled human infection studies (22, 23). Analyses showed that the antibodies generated by these patients recognized gYghJ more effectively than the non-glycosylated protein variant. Importantly, a high proportion of YghJ-specific serum IgA antibodies target epitopes specific to the gYghJ variant. Collectively, these data indicate that carbohydrates are included in a significant part of the epitopes and hence play an important role in the host immune response during infection, but more importantly, during the prevention of infection by vaccination.

For the optimal design of a potential vaccine, it is desirable to understand which are the most immunogenic parts of a protein and, if not all, which antibodies confer protection or resistance against an infection. In most cases, B-cell and T-cell epitopes are predicted by algorithms; however, as recently stated, these are rarely confirmed by actual measurements (24). Alternatively, peptide arrays may be used, but these cannot identify epitopes with conformations induced by the proteins’ secondary or tertiary structures, for example, cysteine-constrained loops or the overall folding of proteins. It is often attempted to compensate for this by using additional setups involving different variants of looped peptides or other modifications (25–27); however, such arrays can become rather large and expensive, and require larger volumes of analytes. Considering additional post-translational modifications, such as glycosylation, the number of variants and limitations in chemical synthesis would require an immense number of epitope variations to be used in assays.

Therefore, we used a novel method based on statistical evaluation of phage display for the identification and comparison of epitopes and mimotopes of anti-YghJ antibodies in the sera of immunized rabbits, pigs, and convalescent patients. The epitope fingerprinting method described recently for allergy-related epitopes (28–30) allows the identification of multiple antibody epitopes directly from serum samples. The naïve random peptide library used in this approach renders not only naïve sequences but also amino acid variations that indicate cross-reactivity. It also allows the *de novo* identification of mimotope motifs from a pool of selected sequences, which might be glycosylation-specific when comparing data from differently immunized animals and patients.

Here, we describe for the first time a large set of peptide epitopes and mimotopes covering the entire YghJ protein, as they are recognized by the immune systems of different species and the role of glycosylation in the development of a stronger immune response.

## 2 Materials and methods

### 2.1 Antigen purification

#### 2.1.1 Glycosylated YghJ (gYghJ) production

To isolate gYghJ, a 3xFLAG epitope tag was added to the *yghJ* gene on the UTI89 chromosome as described in (22). In brief, a PCR product, generated using pSUB11 as a template and the primers GPV122+GPV123 (see **Table 1**), was electroporated into arabinose-induced *E. coli UTI89/pKD46* (31). This generated GPV stain #67. Transformants were selected on LB agar plates containing 40 µg/mL kanamycin. Primer GPV124+GPV121 was used to identify UTI89 *yghJ::*3xFLAG*::kan* clones. Correct insertion was verified by sequencing. Protein purification was done exactly as described in (22).

**Table 1:** Primer names and sequences used in this study.

Primer name	Sequence
GPV 97	TAGCTAGCTCTAGATTACTATTTATCGTCGTCATCTTTG
GPV 121	GCTTATTTTTGACTGCGTACTCG
GPV 122	AAACCGGAAAAAGGGCCGGAAACCATTAACAAGGTTACCGAGCATAAGATGTCTGCCGAGGACTACAAAGACCATGACGG
GPV 123	TTGACCCGATGCGCCTTATATCATGCCGGATGCGGCGTGAACGCCTTATCCGGCCTACAGGCCATATGAATATCCTCCTTAG
GPV 124	CAAACAGTGGTATCCAGATGGTG
GPV 125	ACTTAGATTCAATTGTGAGCCACCATAAGGAGTTTTATAAATGAATAAGAAATTTAAATATAAGAAATCG
GPV 126	TAGCTACTCGAGGGCAAAAAGAGTGTTGACTTGTGAGCGGATAACAATGATACTTAGATTCAATTGTGAGCCACCAT
GPV 127	TCGTTAATATCATCCGGCTTCAT

#### 2.1.2 Non-glycosylated YghJ (ngYghJ) production

A plasmid for production of ngYghJ-3xFLAG was generated essentially as described by Thorsing et al. (2021) (23). In three steps, the UTI89 *yghJ* 3xFLAG sequence was cloned into the expression vector pXG-0 (32), creating pGPV108. First, a DNA fragment containing an IPTG-inducible promoter in front of *yghJ* 3xFLAG was amplified using primers GPV125 + GPV97 and chromosomal DNA from GPV strain #67. In the second PCR step, a XhoI restriction site was added to the DNA fragment by using the primer GPV126. Finally, the PCR product was digested with XhoI and XbaI and ligated into the expression vector pXG-0 to generate plasmid pGPV108. The pGPV108 was transformed in pXG-0 and ngYghJ was purified exactly as described in (22).

### 2.2 Animal sera

Immunization of rabbits and preparation of sera were carried out by Seramun Diagnostica GmbH (Spreenhagener Str. 1, 15754 Heidesee, Germany; permit 2347-15-2023-20-E, LAVG Brandenburg) and Pacific Immunology Inc. (1672 Main St. Ste. E #171, Ramona, CA 92065, USA; USDA Lic No 93-R-0283, NIH OLAW A4182-01). Rabbits were immunized with either gYghJ or the ngYghJ. Rabbits were bled and sera were used for the phage display experiments and array experiments as well. Female pigs (Landrace x Yorkshire) 12–15 weeks old were either vaccinated with gYghJ (*n* = 19) or infected with the UPEC strain UTI89 directly in the bladder (*n* = 17) as part of another study with another primary objective (33). Serum samples were collected for the different experiments using a recently described approach (33). The pig experiments were performed according to the European Union Directive 2010/63/EU on the protection of animals used for scientific purposes, and approved by the Danish Animal Experiments Inspectorate, license number: 2021-15-0201-00821.

Total pig IgG, using 40 mL of serum as input, was purified essentially as described by Fishman and Berg (34). The isolation of pig IgG antibodies specifically recognizing gYghJ was accomplished in two steps. First, 2 mg of ngYghJ was biotin tagged (Thermo Scientific catalog number: 21336) and immobilized on a 1-mL Cytiva HiTrap Streptavidin column. In the second step, 15 mg of Protein A-purified IgG antibodies was passed over the ngYghJ column. The run through containing antibodies recognizing gYghJ as well as IgG unspecific for YghJ was collected and loaded onto a 1-mL Cytiva HiTrap Streptavidin column where 2 mg of biotin-tagged gYghJ had been immobilized. After a washing step, IgG antibodies specifically recognizing gYghJ were eluted using a 0.1 M Glycine buffer (pH = 3). Eluate was pH adjusted to 7.5 and buffer was exchanged against PBS.

### 2.3 Patients’ sera

The sera for the microarray tests were provided by Hospital of Sankt-Georg, Leipzig, Germany. The registry number for the approval by the local ethics committee (Saxon Chamber of Physicians) is EK-BR-101/22-1, Odense University Hospital, Odense, Denmark, approved by research ethics committee of the region of Southern Denmark (S-20200161 KH/csf and Acadre 20/46530); in this study, participants were informed by telephone and in the consultation about the trial, including its risks and disadvantages. After consideration, they provided their written informed consent to participate in the trial.

All patients had recent or ongoing UTI caused by bacteria, all confirmed and characterized by the hospitals’ microbiological laboratories.

### 2.4 Peptide-phage display experiments

To investigate antibody’s binding patterns to YghJ antigen, a statistical peptide phage display approach was applied on serum/antibody samples as described previously (28). By this technique, immunoglobulins of all isotypes were immobilized since no purification step was performed on serum samples. A special naïve peptide phage display is used in the selection experiments (28) and only two selection rounds were applied. Selections from the ENTE-1 peptide phage display library were performed on Dynabeads^®^ Protein A (ThermoFisher Scientific). For each sample, 50-μL beads were added to 20 μL of serum in 100 μL of 0.1% v/v Tween^®^ 20 in PBS (pH=7.6) for 1 h and then washed twice with wash buffer (0.1% v/v Tween^®^ 20 in PBS, pH=7.4). After washing, the beads were resuspended in 200 μL of PBS (pH=7.4). Coupled beads (100 μL) were incubated for 2 h with 4.0 × 10^11^ cfu (respectively 1,000-fold the cfu output from the first selection round) in 1 mL of wash buffer containing 2% w/v BSA. Samples were washed 5× with 1 mL of 0.1% v/v Tween^®^ 20 in PBS for the first round and 5× with 1 mL of 0.5% v/v Tween^®^ 20 in PBS for the second round. The washed beads with bound phage particles were added to a bacterial culture. Phage rescues have been described. Pooled DNA of the recovered phagemids from the first and second selection round was subjected to NGS in an Illumina MiSeq as described. Oversampling and thus excessive data collection are not necessary, since, in this case, the library design based on trinucleotide synthesis gives a strict framework of allowed nucleotides. This allows detection and removal of sequences with potential sequencing errors, after low-quality sequences are removed and the back and forward runs are combined using PEAR and processed applying Trimmomatic (EMBOSS software package). Finally, datasets from each sequencing run were then cured from sequencing errors and other artifacts as described when read into the latest version of the LibDB software (Epitopic GmbH, Leipzig, Germany). Any sequences deviating from the library codon structure are sorted out in this procedure because they potentially contain even additional reading errors by the sequencer.

#### 2.4.1 Analysis of enriched YghJ epitope motifs from sample datasets

As the first step for each dataset, the statistics of all motifs in the datasets are calculated. The statistical value of occurrence is calculated versus the amino acids expected by design of the library. Since the starting library has reproducible and predictable statistical distribution, the amino acids in each position of a 16-mer random sequence, it can be expected that any enrichment is caused by the selection experiment. Special software and a MySQL database allow one to retrieve all peptide sequences containing a specific motif and further analysis by alignment can reveal potential similarity beyond a central motif. Finally, peptides were selected based on the individual alignment results from both the naïve sequence and strongly enriched phage-displayed motifs, in particular when several variants of these are surrounded by two cysteines or other conserved amino acids.

Primary *in silico* analysis focused on comparing the enrichment values from YghJ motifs among datasets obtained from all sample datasets. Epitope motifs enriched in at least two to three serum samples were considered for further analysis.

### 2.5 IgG-binding measurements using peptide microarrays

#### 2.5.1 Selection of peptide epitopes/mimotopes for spotting on microarray slides

Peptides were derived from either the native YghJ antigen sequences (referred as *epitopes*) or *mimotopes* obtained from phage-displayed sequences in selection experiments. Chosen peptides and their source are listed in the Appendix.

#### 2.5.2 Peptide microarrays

Peptides were purchased from peptides&elephants GmbH (Hennigsdorf, Germany). All peptides had a C-terminal ebes-ϵ-azido-Lys linker, so they could be printed and immobilized as triplicates on DBCO-coated glass slides using click chemistry. The surfaces of these slides had been prepared via silanization. A stock solution of 50 mg/mL DBCO-amine in DMF (molecular sieve dried) was prepared for this purpose. The slides were incubated with 120 μL of DBCO solution (0.25 mg/mL) and incubated overnight in the dark at room temperature. They are then washed with EtOH and centrifuged dry. Slides are stored at −20°C.

The slides contained eight identical peptide arrays, each one intended to contain one serum sample. Each cluster contained 61 peptide epitopes spotted in triplicates. We measured IgG binding to peptide epitopes.

Each slide was blocked for 1 h at 4°C in array buffer (PBS containing 0.1% v/v Tween^®^ 20 and 1% w/v Casein, pH 7.4), which was also used for all further solutions. The slides were incubated for 2 h at RT with patient serum diluted 1:50, washed twice (PBS containing 0.1% v/v Tween^®^ 20, pH 7.4), incubated for 1 h at RT in a solution of mouse anti-human IgG antibody (1:5,000; Abcam, Cambridge, UK) and washed twice again. Antibody binding was detected by incubating the array for 1 h at RT with a secondary Cy5-labeled goat anti-mouse antibody (1:5,000; ThermoFisher Scientific). Finally, the slides were washed again twice and fluorescence was measured in a microarray reader at 10 μm resolution using a laser at 532 nm with 25% power/PMT Gain 600 and 635 nm with 25% power/PMT Gain 600 (Genepix 4300; Molecular Devices, San Jose, CA, USA).

#### 2.5.3 Determination of spot intensities

The first step is the detection of the *a priori* known grid (GAL file) in the image, as the grid may occur rotated or shifted. Therefore, we used an image correlation technique that observes the whole grid at once to estimate the exact location of the grid. Afterward, the location of each block underwent another more precise correction to address for block-specific rotation and translation. This step was corrected if necessary and validated before continuing with the analysis.

With the known position of each spot, we used a segmentation approach that combines a seeding threshold and a masking threshold with a geodesic dilation to distinguish between foreground and background signals. This shall result in one or a few connected segments that surround the exact shape of the spot. To address single-pixel inaccuracies, the shape gets blurred with morphological binary operations.

In the last step of image analysis, we extracted information regarding the fluorescence intensity and shape of the segmentation. Information about the shape of a segment was mainly used to filter invalid segments due to artifacts.

As a reliable and robust method to decide for a positive or negative spot result, we used the total count of all pixels in the spot after subtracting the background per pixel, summarizing all intensities in a spot segment, and subtracting the median of the block background for every pixel.

The raw total fluorescence signal intensity of triplicates was used to calculate the upper/lower quartile. The difference between upper and lower quartiles was multiplied with 1.5. This number was added to the upper quartile and subtracted from the lower quartile. All raw fluorescence signal intensity not located in this calculated range was removed. The adjusted raw fluorescence signal intensity was calculated as a multiple of the background (water).

## 3 Results

### 3.1 Identification of epitopes

Epitopes were identified using the statistical peptide phage display method described previously. The method allows the identification of binding peptides, that is, epitopes, by searching NGS datasets for enriched motifs in all sequences of the NGS sequence pool obtained after one or two rounds of selection. This avoids repeated selection rounds, and the method is therefore generated by NGS large datasets consisting of pools of sequence variants for most antibody epitopes. Such are usually lost in repeated selection rounds. We identified 48 potential epitopes using sera from rabbits immunized with either gYghJ or ngYghJ, pigs immunized with gYghJ, pigs infected directly in the bladder with UPEC, and patients with a recent history of UTI caused by *E. coli*. The method applied allows the restriction of the peptide epitopes to a minimal size comprising the amino acids that are recognized by antibodies of different sera, as can be judged from the alignment of multiple similar enriched sequences.

**Figure 1:**
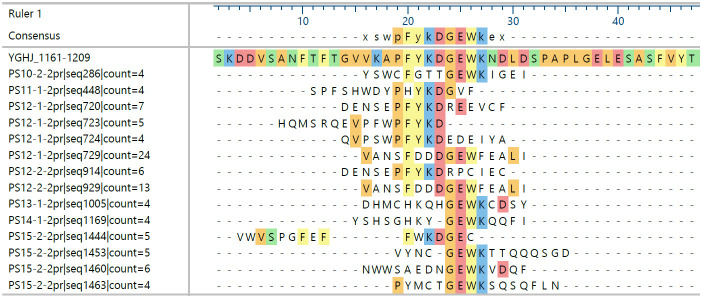
Sequences sharing motifs of the epitope motif 1178-PKFYKDGEWK enriched by binding to different patient sera’s antibodies. Patients (PS) 10, 13, 14, and 15 seem to form a group different from 11 and 12. Displayed are only those sequences found at least four times and sharing at least five amino acid identities in the alignment selected from a total of 2,815 sequences from 12 datasets with an average frequency of 2; the naïve library has an average of ca. 1.1 for all sequences in a comparable dataset.

Overall, all identified epitopes are from regions of high similarity between YghJ and bacterial pathogens sharing 50% or more sequence identity or at least apparent structural similarities, for example, Cys or charge patterns. Nevertheless, although individual immune systems share epitope sites, there are distinct differences between the essential amino acids recognized in several cases. **Figure 1** shows an example of how this is reflected in sequences with enriched motifs of one epitope using sera from convalescent patients.

**Figure 2:**
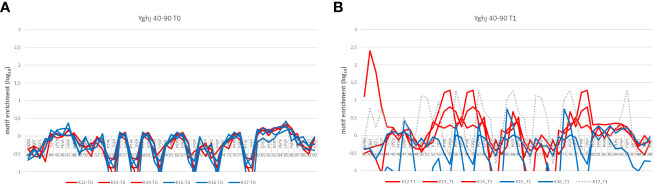
Differences in the enrichment pattern of epitope motifs between gYghJ and ngYghJ comparing sera before (t0) and after (t1) immunization. **(A)** Prior to immunization, the sera does not contain YghJ-specific antibodies. **(B)** Rabbits K12, K13, and K14 (red lines) have been immunized with the glycosylated antigen and strongly enrich sequences containing proline-rich motifs. This enrichment is not found in the Rabbits K15 and K16 immunized with non-glycosylated antigen (blue line). Rabbit K17 was also immunized with non-glycosylated antigen (hatched line), but probably an additional contact to another (glycosylated) antigen led to the different enrichment pattern.

Statistical analysis of enriched sequence motifs in the datasets alone can be used as an indicator to show differences between gYghJ and the non-glycosylated protein variant. The region 44–90 aa is a poly proline-rich domain within the protein, and rabbits show recognition patterns depending on the antigen used for immunization (**Figure 2**). None of the rabbit data showed enrichment prior to immunization. Rabbits immunized with the glycosylated protein showed high enrichment in this region. Rabbits immunized with ngYhgJ also showed weak enrichment in this region, but the recognition pattern was different (**Figure 2B**). One exception was rabbit K17, which also showed high enrichment in this region. It is likely that this rabbit was infected with Enterobacteria prior to or more likely during immunization. The statistical enrichment of 4-mer motifs in this region in NGS datasets after two rounds of selection for this animal is shown in **Figure 2B**.

These differences in sequence datasets have been used to identify identical or similar peptides that have been enriched in rabbits immunized with the glycosylated antigen. Eight peptides were selected as potential peptide mimotopes based on their selective enrichment in the datasets. Two of these share homology with a peptide containing the motif YghJ 585-SKGE, which is apparently a signature for glycosylated YghJ antigens. Strongly enriched phage sequences showed a preference for a Trp N- or C-terminal motif and two well-enriched peptides were selected (**Table 2**).

**Table 2:** Mimotopes selected on specific enrichment and similarity to peptide epitopes.

Peptide Code	Sequence
YghJ Y-585-N1	VN**SKGE**STLSGD
YMi-06	KN**SKGE**EQE **W** Q
YMi-07	C **W** AN**SKGE**EQGTC

In bold the essential binding motif SKGE accompanied by Trp (bold underlined) in mimotopes selected on sera from rabbits immunized with gYghJ.

### 3.2 Results from array measurements

**Figure 3:**
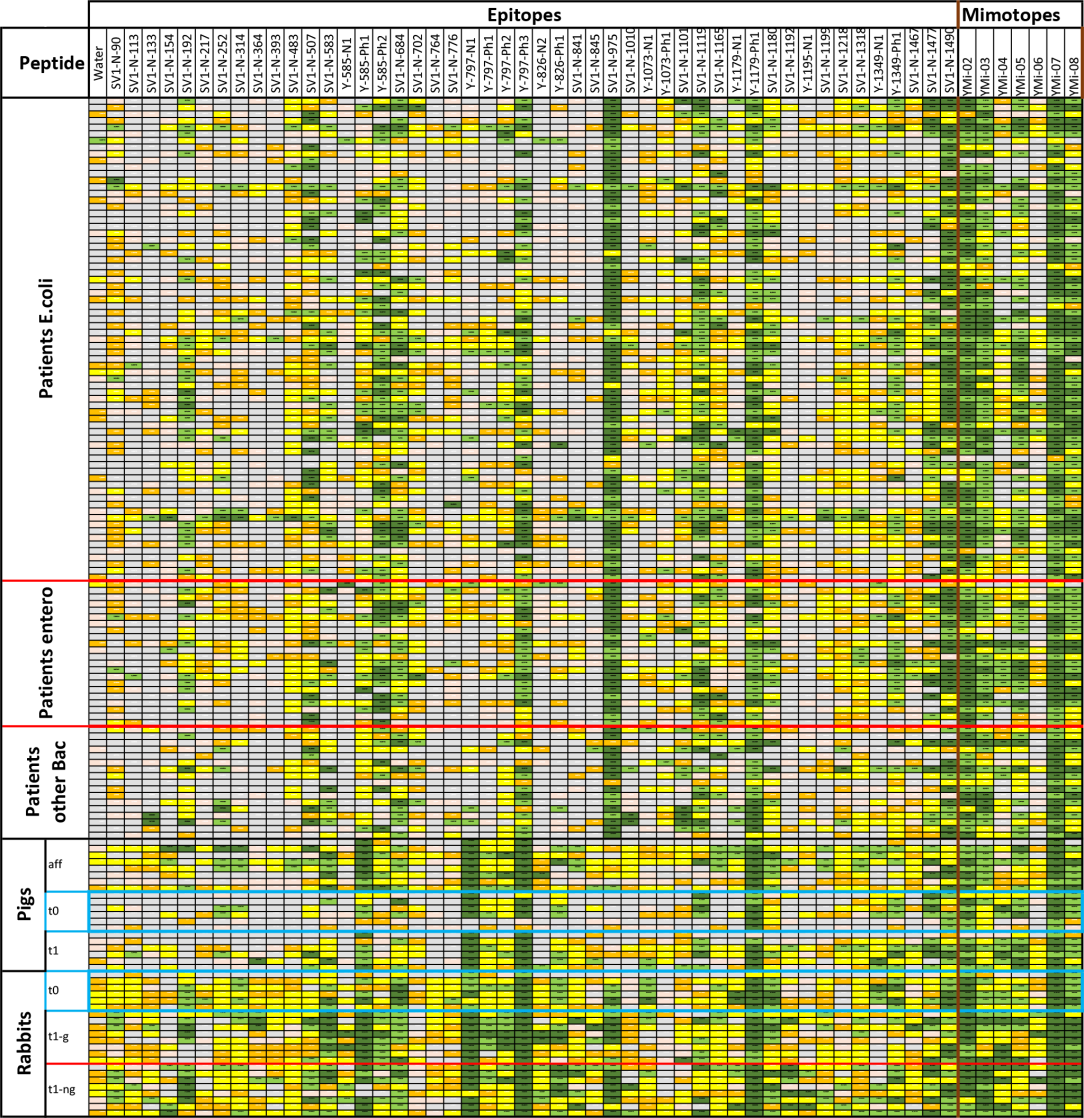
Results from screening sera from UTI patients with confirmed infections, pigs either vaccinated or infected directly in the bladder, and immunized rabbits that have been tested in an epitope peptide array with triplicates. The signal cutoffs are signal over background (empty spots). Color codes are signal over background; >100× (dark green), >20× (light green), >4× (yellow), >2× (orange), 2× and less (gray); no data due to too high error, printing errors in the array or not at least values from 2 spots. Blue boxes: Animal sera taken prior to immunization.

Based on the epitopes identified using rabbit and convalescent patient serum, peptides were synthesized and spotted as microarrays on glass slides. Sera from immunized rabbits, immunized and infected pigs, and patients were used in the analysis (**Figure 3**). Rabbits and pigs were immunized with different antigens (glycosylated/non-glycosylated; see above).

**Figure 4:**
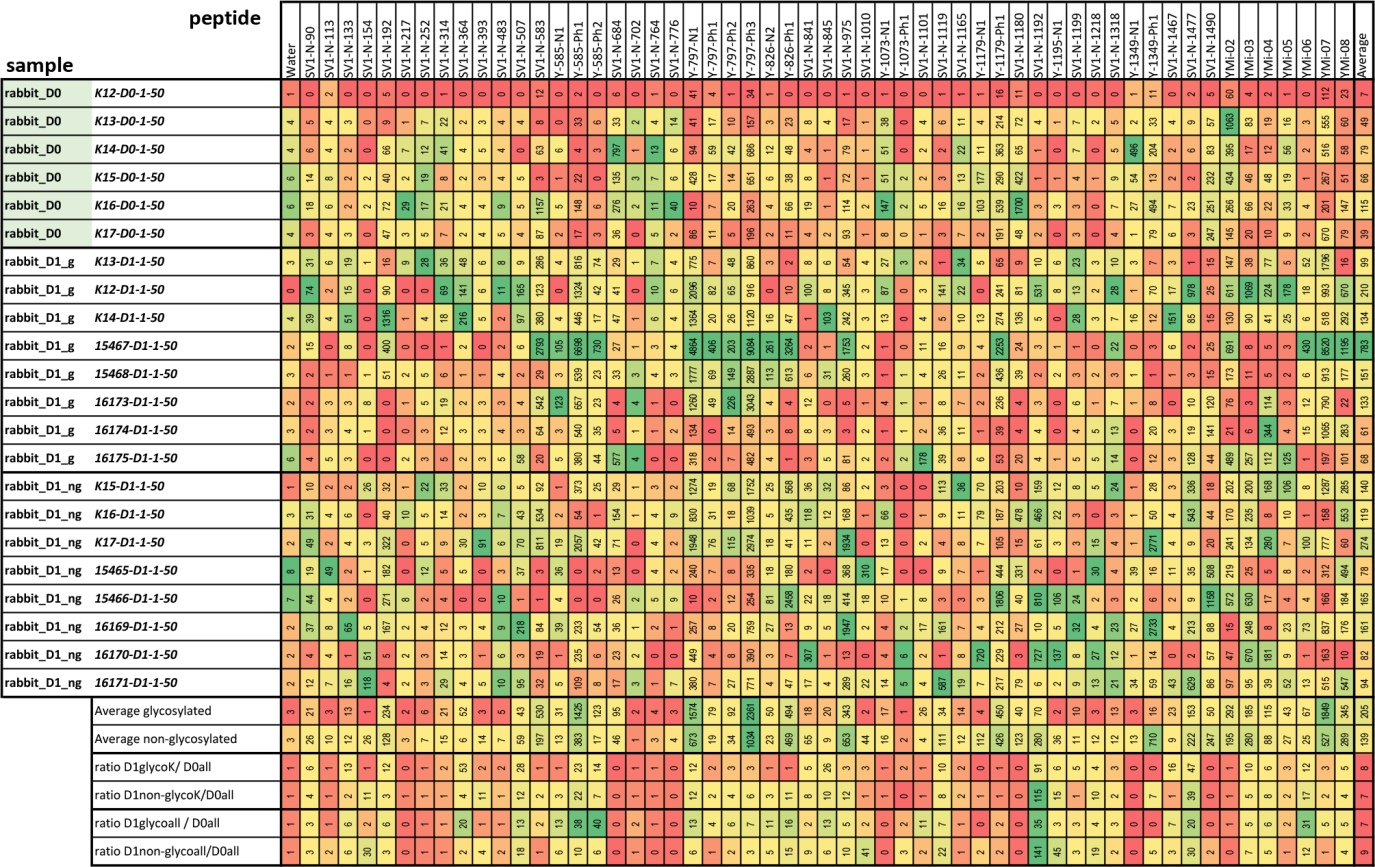
Absolute values measured in peptide microarrays with rabbit sera prior to immunization (D0) and after immunization with the glycosylated and non-glycosylated antigen. The color code is adjusted for each peptide individually from maximal (green) to lowest (red) value in direct comparison. The lower lines compare the signals for each peptide giving the average and the ratios between sera pre- and post-immunization with the different antigens. Only for the rabbits labeled “K” was a pre-immune serum taken; hence, the different ratios were calculated at the end of the table.

All identified peptide epitopes and phage display-derived peptide mimotopes showed IgG binding from at least several sera (**Figure 3**). The signal varies with the individual immune response, particularly because the short sequences used are likely to have different affinities for individual antibody clones. Signal intensities vary greatly with different species, and a direct comparison can only be performed with the results from immunized rabbits’ sera (**Figure 4**). Although at least one rabbit had contact with a YghJ-like antigen, there was a tendency for rabbits prior to immunization to have much lower signals in the peptide microarray data than after immunization. The glycosylated vaccine caused a different pattern and higher signal strength compared to the non-glycosylated protein.

Comparing different species, the divergence of the individual epitope patterns was high, both between UTI patients and infected or immunized animals (**Figure 3**). However, there was no general difference in the antigen epitopes recognized by the sera of animals and UTI-patient sera. Surprisingly, the epitope pattern did not depend on the bacterial species causing ongoing infection. We compared the selected epitope peptides with different known AcfD proteins, and all were located in highly conserved areas (see **Supplementary Data**).

Sera taken before the treatment of both pigs and rabbits show a significant number of different positive epitopes. This is most likely due to common exposure to the highly conserved antigen among the *E. coli* pathogens. However, upon exposure to vaccination or infection, antibodies against additional epitopes or stronger responses are observed. In the initial studies, reference sera were considered; however, the first tested epitopes were recognized by both non-immunized and immunized rabbits. Antibodies against the antigen epitopes can probably be found in all sera samples of mammals regularly exposed to Enterobacteria. This applies even to some rabbits, which are usually not exposed to Enterobacteria, as opposed to pigs and humans.

The sera from pigs generally showed lower levels of antibodies against the antigen. Nevertheless, by using affinity-purified antibodies, it was revealed that these animals also have the entire spectrum of antibodies.

**Figure 5:**
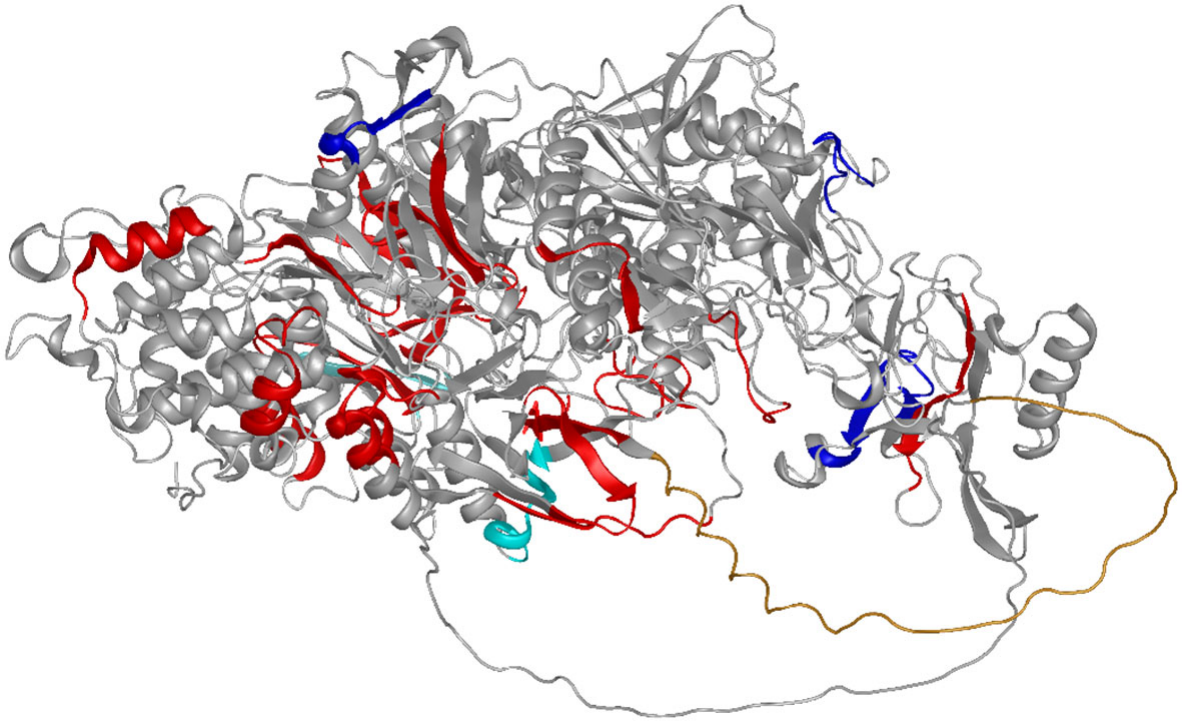
Summarizing the results of this study: The epitopes are shown on the AF-model for a related YghJ (E3PJ90) (35–37). Epitopes are shown in red, with the exception of the epitopes positively (dark blue) or negatively (light blue) influenced by glycosylation. The N-terminal proline-rich loop (compare **Figure 3**) protrudes as an undefined orange tube structure towards the reader.

The effect of glycosylation on the antibody signals in the arrays was observed in rabbits for several epitopes (**Figure 4**). This has been described earlier for full-length YghJ. Some of these epitopes show extreme enrichment and are likely to be mimotopes of glycosylation sites, that is, epitopes with a hydrophobic pattern mimicking a carbohydrate structure (**Table 1**). The summary for the identified epitopes and locations in the protein are shown **Figure 5**.

## 4 Discussion

This study is the first attempt to characterize and compare the immune responses to a bacterial antigen in infected patients and immunized animals at the epitope level. Overall, the array revealed that the immune system of vaccinated rabbits recognizes the same epitopes as antibodies present in the serum of convalescent patients. In addition, pigs vaccinated with gYghJ or infected directly in the bladder with *E. coli* raised antibodies against almost all the epitopes observed in rabbits and humans. This epitope analysis indicates that the vaccination of rabbits and pigs raises antibodies that translate well into the human immune system.

Since it is likely that humans and pigs have been exposed to the antigen by contact with Enterobacteria before immunization/infection, at least by intestinal bacterial strains, there is a pre-existing and individual B-cell response to the antigen/pathogen. Most rabbits in this study and a previous study were apparently less affected by this effect, which may be explained by a different microbiome. However, some rabbits have generated antibodies against the antigen or a closely related antigen. There were too few animals involved in this study to allow for more detailed analysis.

The analysis of patient data suggests that patients who usually undergo multiple infections (38) develop a rather heterogeneous response caused by exposure to a variety of homologous proteins of different pathogenic strains.

### 4.1 Comparison to other methods

In comparable studies, the epitopes for T and B cells are predicted by algorithms and are often not precisely specified to individual amino acid residues (39, 40). Even peptide arrays will only locate the rough position of an epitope and can be material and cost intensive when screening hundreds of patients and potentially several homologous proteins. The method used here compares individual immune responses *in silico* and allows for the identification of key residues commonly recognized by the adaptive immune response. Surprisingly, short peptides not only are recognized by different mammalian species, but also unveil cross-reactivities of antibodies to antigen epitopes that do not share all amino acids, as a retrospective alignment of YghJ domains showed (provided in the **Supplementary Data**). All identified YghJ peptide epitopes were aligned to AcfD proteins. Within the small variations among the majority of these proteins, there is not a single case where cross-reactivity could be excluded. On the other hand, it could be concluded that immunization with *E. coli* YghJ boosts the immune response to most AcfD domains of pathogens involved in UTI.

### 4.2 Lack of controls

YghJ is highly conserved, and any contact with pathogenic Enterobacteriaceae carries the risk of exposure to homologues (21). All animals, and probably not only mammals, whether they experienced UTI or not, are therefore likely to develop these antibodies at some stage over a longer lifetime. This may, in particular, be true for the pigs used in the studies, since they are born and raised in facilities where diarrhea-causing enterotoxigenic *E. coli* is often a problem. Rabbits in their relative short lifetime and a rather divergent microbiome could be expected to be less prone to such pre-immunization (41). However, some of the rabbits used in the experiments showed epitope patterns that were comparable to those after immunization with glycosylated antigen. It remains unclear, however, whether this is a general response driven by bacterial glycosylation or by exposure to a natural source of YghJ. The peptide-directed response is more likely to occur in the latter case. Although previous studies have not detected such antibodies using Western blotting, the serum concentration in microarray experiments is much higher, and the sensitivity and dynamic range are orders of magnitude higher when using small spots and a fluorescent dye-based detection system.

### 4.3 Comparison to recent results (glycosylation)

Recent studies have shown that gYghJ is significantly better recognized by serum antibodies isolated from convalescent patients than by non-glycosylated protein variants (22, 23). Moreover, a substantial amount of serum anti-YghJ-IgA antibody binds exclusively to the glycosylated protein. Using rabbits, we can now determine the individual epitopes in the glycosylated protein. In these animals, comparing vaccination with gYghJ to ngYghJ the glycosylation causes different or enhanced immune responses. For example, the N-terminal region 40–90 shown in **Figure 2B**, as well as the epitopes 364, 585, and 797 listed in **Figure 4**, showed responses that were up to 40-fold increased. According to our observations, the complete absence of enriched motifs in the N-terminal region is a reliable indicator of non-glycosylated antigens and a lack of previous immunization with natural YghJ. Conversely, array analysis also showed that vaccination with ngYghJ resulted in stronger responses towards epitopes 154, 1,192, and 1,195. When examining the responses against these six epitopes in pigs before and after bladder infection caused by *E. coli*, 585 and 797 were strongly recognized, whereas 154, 1,192, and 1,195 only resulted in weak signals. The absence of a signal in infected animals indicates that a vaccine based on the ngYghJ protein may raise individual sets of immunologically irrelevant antibodies. This view is supported by a comparison of patient responses to the six epitopes. Sequences 585 and 797 were well recognized by the antibodies throughout the patient group, whereas 154, 1,192, and 1,195 hardly gave rise to a signal in any of the individuals. Based on these results, it is unlikely that a vaccine lacking these modifications would confer the desired protection against the pathogen. In this respect, glycosylation is even more important than has recently been discussed.

When searching for sequences containing motifs that were more strongly enriched after immunization with the glycosylated protein, we expected to find mimotopes or short naïve epitopes with adjacent structures similar to a carbohydrate structure. The immune response to the identified sequences came initially as a surprise. In some cases, a strong antibody response exists even in pre-immune sera. However, since bacterial glycosylation patterns are also bound to structural requirements, these mimotopes are likely to present a general immune response to glycosylation in many other bacterial proteins.

## Data availability statement

The original contributions presented in the study are included in the article/**Supplementary Material**, further inquiries can be directed to the corresponding author/s.

## Ethics statement

The studies involving human participants were reviewed and approved for Hospital of Sankt-Georg, Leipzig, Germany by the local ethics committee (Saxon Chamber of Physicians) is EK-BR-101/22-1; and for Odense University Hospital, Odense, Denmark, by research ethics committee of the region of Southern Denmark (S-20200161 KH/csf and Acadre 20/46530), in this study participants were informed by telephone and in the consultation about the trial, including its risks and disadvantages. After consideration, they provided their written informed consent to participate in the trial. The animal study utilizing pig experiments was reviewed and approved by Danish Animal Experiments Inspectorate, license number: 2021-15-0201-00821.

## Author contributions

MS: Conceptualization, Formal analysis, Funding acquisition, Methodology, Project administration, Software, Writing – original draft, Writing – review & editing. KK: Data curation, Formal analysis, Investigation, Methodology, Software, Visualization, Writing – review & editing. ND: Funding acquisition, Investigation, Methodology, Project administration, Writing – review & editing. AB: Conceptualization, Formal Analysis, Funding acquisition, Investigation, Methodology, Project administration, Resources, Supervision, Writing – original draft, Writing – review & editing. MP: Formal analysis, Software, Writing – review & editing. RW: Resources, Writing – review & editing. AK: Investigation, Resources, Writing – review & editing. EE-F: Methodology, Resources, Writing – review & editing. KS: Investigation, Methodology, Resources, Writing – review & editing. TA: Investigation, Methodology, Resources, Writing – review & editing. KA: Resources, Writing – review & editing. LL: Resources, Writing – review & editing.
