# Supplementary material for: Glycosylation of bacterial antigens changes epitope patterns
Source: Front Immunol. 2023 Oct 26;14:1258136. doi: 10.3389/fimmu.2023.1258136 (PMC10637626; doi:10.3389/fimmu.2023.1258136)
Supplement: Supplementary file 1 [file DataSheet_1.pdf]

Supplement „**Glycosylation of bacterial antigens changes epitope patterns**”

Contents

		**Page**
**List of peptide sequences**		2
**Alignments with AcfDs**	SV1-N-90	3
	SV1-N-113	3
	SV1-N-133	4
	SV1-N-154	4
	SV1-N-192	5
	SV1-N-217	5
	SV1-N-252	6
	SV1-N-314	7
	SV1-N-364	8
	SV1-N-393	8
	SV1-N-483	9
	SV1-N-507	9
	SV1-N-583	10
	Y-585-N1	10
	SV1-N-684	11
	SV1-N-702	12
	SV1-N-764	13
	SV1-N-776	13
	Y-797-N1	14
	Y-826-N2	15
	SV1-N-841	16
	SV1-N-845	16
	SV1-N-975	17
	SV1-N-1010	18
	Y-1073-N1	19
	SV1-N-1101	19
	SV1-N-1119	19
	SV1-N-1165	20
	Y-1179-N1	20
	SV1-N-1180	20/21
	SV1-N-1192	21
	Y-1195-N1	21
	SV1-N-1199	21
	SV1-N-1218	21
	SV1-N-1318	22
	Y-1349-N1	23
	SV1-N-1467	24
	SV1-N-1477	24
	SV1-N-1490	24

List of peptide sequences			
Code	N-Terminus	Sequence	C-Terminus
SV1-N-90	Ac-	PTKTGYLTLGGSQ	ebes-epsilon-azido-Lys
SV1-N-113	Ac-	SSDGFTFKPGEDV	ebes-epsilon-azido-Lys
SV1-N-133	Ac-	IATFNTQSEAAR	ebes-epsilon-azido-Lys
SV1-N-154	Ac-	VSFSLEDAQELAGS	ebes-epsilon-azido-Lys
SV1-N-192	Ac-	TFSSVVDRARFEKL	ebes-epsilon-azido-Lys
SV1-N-217	Ac-	KKLVNEEVENNA	ebes-epsilon-azido-Lys
SV1-N-252	Ac-	PDLNASFVSANAE	ebes-epsilon-azido-Lys
SV1-N-314	Ac-	GEAISFGIDTFE	ebes-epsilon-azido-Lys
SV1-N-364	Ac-	NTRVVPDDVRKV	ebes-epsilon-azido-Lys
SV1-N-393	Ac-	NGATLDEGDQNV	ebes-epsilon-azido-Lys
SV1-N-483	Ac-	GNARGQAVVNIS	ebes-epsilon-azido-Lys
SV1-N-507	Ac-	DKNYWLAFGEK	ebes-epsilon-azido-Lys
SV1-N-583	Ac-	YSWGGGVNSKGE	ebes-epsilon-azido-Lys
Y-585-N1	Ac-	VNSKGESTLSGD	ebes-epsilon-azido-Lys
Y-585-Ph1	Ac-	GCLHNSKGECV	ebes-epsilon-azido-Lys
Y-585-Ph2	Ac-	CNQNSKGECTQ	ebes-epsilon-azido-Lys
SV1-N-684	Ac-	FEYVTQWGSDPY	ebes-epsilon-azido-Lys
SV1-N-702	Ac-	DTSKPKLTQQDV	ebes-epsilon-azido-Lys
SV1-N-764	Ac-	QGYPDRVRQR	ebes-epsilon-azido-Lys
SV1-N-776	Ac-	RSTPIWVYERYP	ebes-epsilon-azido-Lys
Y-797-N1	Ac-	TIDPNTGEVTWKY	ebes-epsilon-azido-Lys
Y-797-Ph1	Ac-	CQDPNTGEDCE	ebes-epsilon-azido-Lys
Y-797-Ph2	Ac-	ACDPNTGECR	ebes-epsilon-azido-Lys
Y-797-Ph3	Ac-	CDPNTGECRQHEF	ebes-epsilon-azido-Lys
Y-826-N2	Ac-	QEEVEGKQVTRY	ebes-epsilon-azido-Lys
Y-826-Ph1	Ac-	VCEEVEGKCS	ebes-epsilon-azido-Lys
SV1-N-841	Ac-	AFIDEAEYTTEE	ebes-epsilon-azido-Lys
SV1-N-845	Ac-	TTEESLEAAKAK	ebes-epsilon-azido-Lys
SV1-N-975	Ac-	LGFKTFTEFLN	ebes-epsilon-azido-Lys
SV1-N-1010	Ac-	IYGEESSKAG	ebes-epsilon-azido-Lys
Y-1073-N1	Ac-	NPTKWFAGNAQ	ebes-epsilon-azido-Lys
Y-1073-Ph1	Ac-	SGFNPTKWFST	ebes-epsilon-azido-Lys
SV1-N-1101	Ac-	IKSSASVPVTVT	ebes-epsilon-azido-Lys
SV1-N-1119	Ac-	LTGREKHEVALN	ebes-epsilon-azido-Lys
SV1-N-1165	Ac-	DDVSANFTFT	ebes-epsilon-azido-Lys
Y-1179-N1	Ac-	APFYKDGEWKNDLD	ebes-epsilon-azido-Lys
Y-1179-Ph1	Ac-	CFQRYKDGRCLED	ebes-epsilon-azido-Lys
SV1-N-1180	Ac-	FYKDGEWKNDL	ebes-epsilon-azido-Lys
SV1-N-1192	Ac-	DSPAPLGELES	ebes-epsilon-azido-Lys
Y-1195-N1	Ac-	DSPAPLGELES	ebes-epsilon-azido-Lys
SV1-N-1199	Ac-	ELESASFVYTTP	ebes-epsilon-azido-Lys
SV1-N-1218	Ac-	TGGLEQFANDLD	ebes-epsilon-azido-Lys
SV1-N-1318	Ac-	PGATEVANNVLA	ebes-epsilon-azido-Lys
Y-1349-N1	Ac-	TVAPEYLDES	ebes-epsilon-azido-Lys
Y-1349-Ph1	Ac-	HSHVAPEYEKE	ebes-epsilon-azido-Lys
SV1-N-1467	Ac-	GASAYQLPGATE	ebes-epsilon-azido-Lys
SV1-N-1477	Ac-	GASELSFEGGVS	ebes-epsilon-azido-Lys
SV1-N-1490	Ac-	GVSQSAYNTLAS	ebes-epsilon-azido-Lys
YMi-01	Ac-	CQDHDNDYPTFTC	was planned but not synthesized
YMi-02	Ac-	DQQDHDWDYHAF	ebes-epsilon-azido-Lys
YMi-03	Ac-	CFDHDLDYQIGCY	ebes-epsilon-azido-Lys
YMi-04	Ac-	SKDHDQDYYVD	ebes-epsilon-azido-Lys
YMi-05	Ac-	SITNDHDQDYKSF	ebes-epsilon-azido-Lys
YMi-06	Ac-	KNSKGEEQEWQ	ebes-epsilon-azido-Lys
YMi-07	Ac-	CWANSKGEEQGTC	ebes-epsilon-azido-Lys
YMi-08	Ac-	VDHDHDYDVHKY	ebes-epsilon-azido-Lys

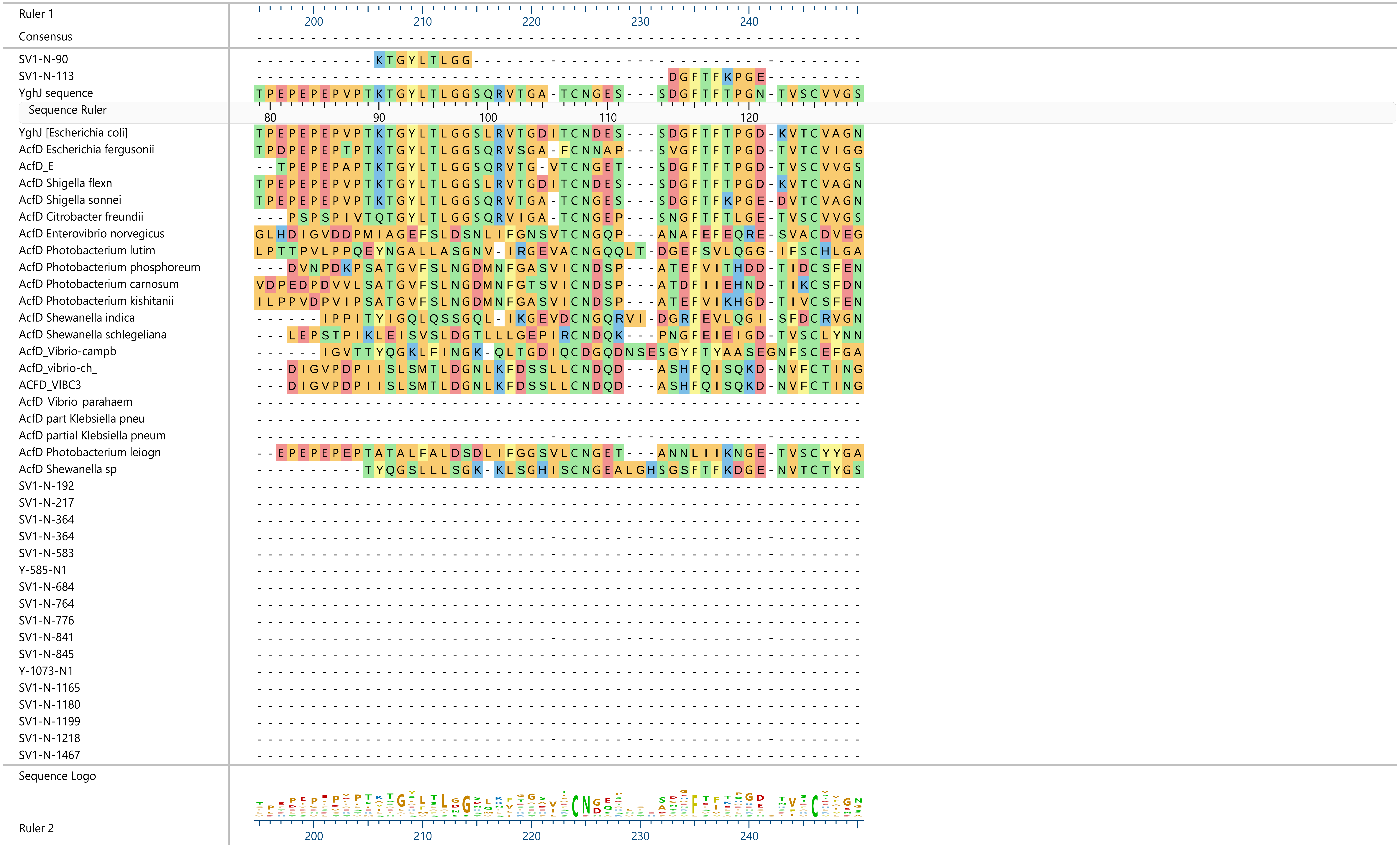
Ruler 1
200 210 220 230 240
Consensus --
SV1-N-90 -----------KTGYLTLGG------------------------------------
SV1-N-113 --------------------------------------DGFTFKPGE---------
YghJ sequence TPEPEPEPVPTKTGYLTLGGSQRVTGA-TCNGES---SDGFTFTPGN-TVSCVVGS
Sequence Ruler
80 90 100 110 120
YghJ [Escherichia coli] TPEPEPEPVPTKTGYLTLGGSLRVTGDITCNDES---SDGFTFTPGD-KVTCVAGN
AcfD Escherichia fergusonii TPDPEPEPTPTKTGYLTLGGSQRVSGA-FCNNAP---SVGFTFTPGD-TVTCVIGG
AcfD_E --TPEPEPAPTKTGYLTLGGSQRVTG-VTCNGET---SDGFTFTPGD-TVSCVVGS
AcfD Shigella flexn TPEPEPEPVPTKTGYLTLGGSLRVTGDITCNDES---SDGFTFTPGD-KVTCVAGN
AcfD Shigella sonnei TPEPEPEPVPTKTGYLTLGGSQRVTGA-TCNGES---SDGFTFKPGE-DVTCVAGN
AcfD Citrobacter freundii ---PSPSPIVTQTGYLTLGGSQRVIGA-TCNGEP---SNGFTFTLGE-TVSCVVGS
AcfD Enterovibrio norvegicus GLHDIGVDDPMIAGEFSLDSNLIFGNSVTCNGQP---ANAFEFEQRE-SVACDVEG
AcfD Photobacterium lutim LPTTPVLPPQEYNGALLASGNV-IRGEVACNGQQLT-DGEFSVLQGG-IFSCHLGA
AcfD Photobacterium phosphoreum ---DVNPDKPSATGVFSLNGDMNFGASVICNDSP---ATEFVITHDD-TIDCSFEN
AcfD Photobacterium carnosum VDPEDPDVVLSATGVFSLNGDMNFGTSVICNDSP---ATDFIIEHND-TIKCSFDN
AcfD Photobacterium kishitanii ILPPVDPVIPSATGVFSLNGDMNFGASVICNDSP---ATEFVIKHGD-TIVCSFEN
AcfD Shewanella indica ------IPPITYIGQLQSSGQL-IKGEVDCNGQRVI-DGRFEVLQGI-SFDCRVGN
AcfD Shewanella schlegeliana ---LEPSTPIKLEISVSLDGTLLLGEPIRCNDQK---PNGFEIEIGD-TVSCLYNN
AcfD_Vibrio-campb ------IGVTTYQGKLFINGK-QLTGDIQCDGQDNSESGYFTYAASEGNFSCEFGA
AcfD_vibrio-ch_ ---DIGVPDPIISLSMTLDGNLKFDSSLLCNDQD---ASHFQISQKD-NVFCTING
ACFD_VIBC3 ---DIGVPDPIISLSMTLDGNLKFDSSLLCNDQD---ASHFQISQKD-NVFCTING
AcfD_Vibrio_parahaem --
AcfD part Klebsiella pneu --
AcfD partial Klebsiella pneum --
AcfD Photobacterium leiogn --EPEPEPEPTATALFALDSDLIFGGSVLCNGET---ANNLIIKNGE-TVSCYYGA
AcfD Shewanella sp ----------TYQGSLLLSGK-KLSGHISCNGEALGHSGSFTFKDGE-NVTCTYGS
SV1-N-192 --
SV1-N-217 --
SV1-N-364 --
SV1-N-364 --
SV1-N-583 --
Y-585-N1 --
SV1-N-684 --
SV1-N-764 --
SV1-N-776 --
SV1-N-841 --
SV1-N-845 --
Y-1073-N1 --
SV1-N-1165 --
SV1-N-1180 --
SV1-N-1199 --
SV1-N-1218 --
SV1-N-1467 --
Sequence Logo
Ruler 2
200 210 220 230 240

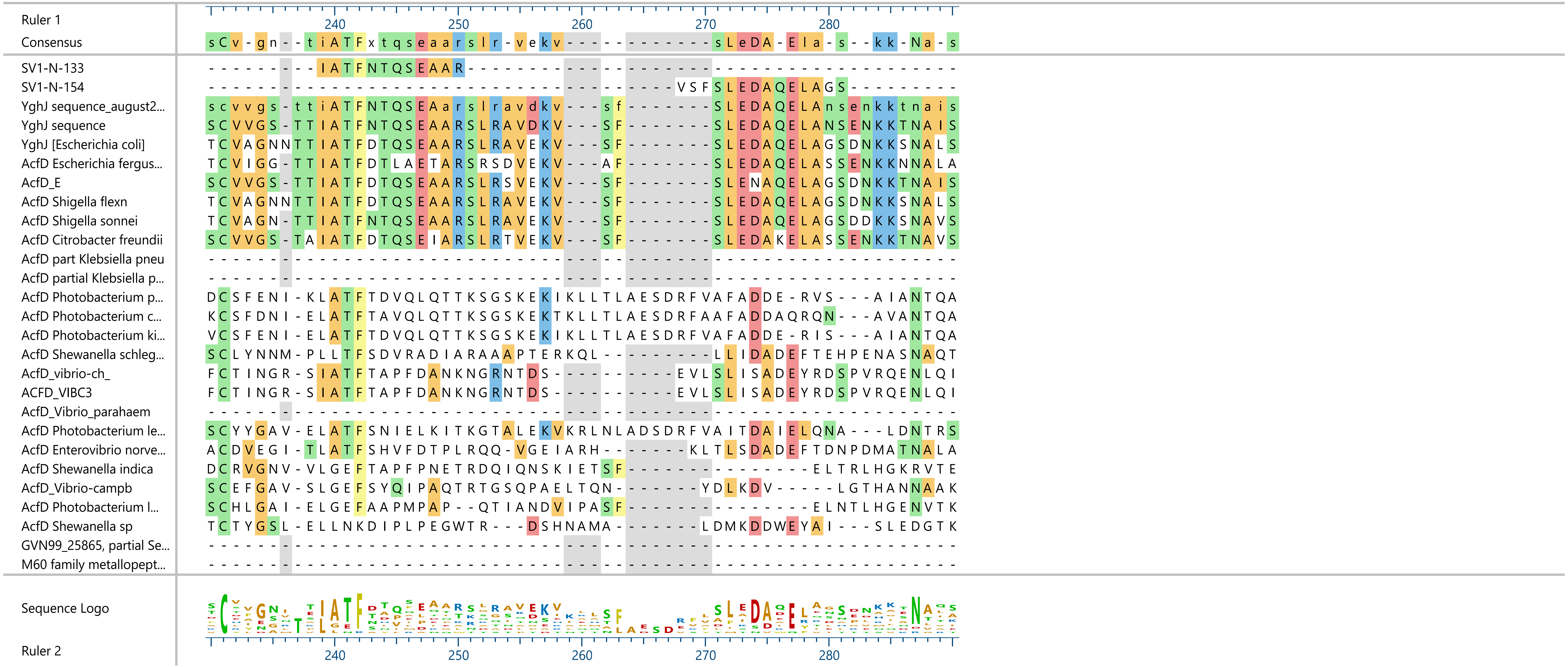
Ruler 1
240
250
260
270
280
Consensus
SV1-N-133
SV1-N-154
YghJ sequence_august2...
YghJ sequence
YghJ [Escherichia coli]
AcfD Escherichia fergus...
AcfD_E
AcfD Shigella flexn
AcfD Shigella sonnei
AcfD Citrobacter freundii
AcfD part Klebsiella pneu
AcfD partial Klebsiella p...
AcfD Photobacterium p...
AcfD Photobacterium c...
AcfD Photobacterium ki...
AcfD Shewanella schleg...
AcfD_vibrio-ch_
ACFD_VIBC3
AcfD_Vibrio_parahaem
AcfD Photobacterium le...
AcfD Enterovibrio norve...
AcfD Shewanella indica
AcfD_Vibrio-campb
AcfD Photobacterium l...
AcfD Shewanella sp
GVN99_25865, partial Se...
M60 family metallopept...
Sequence Logo
Ruler 2

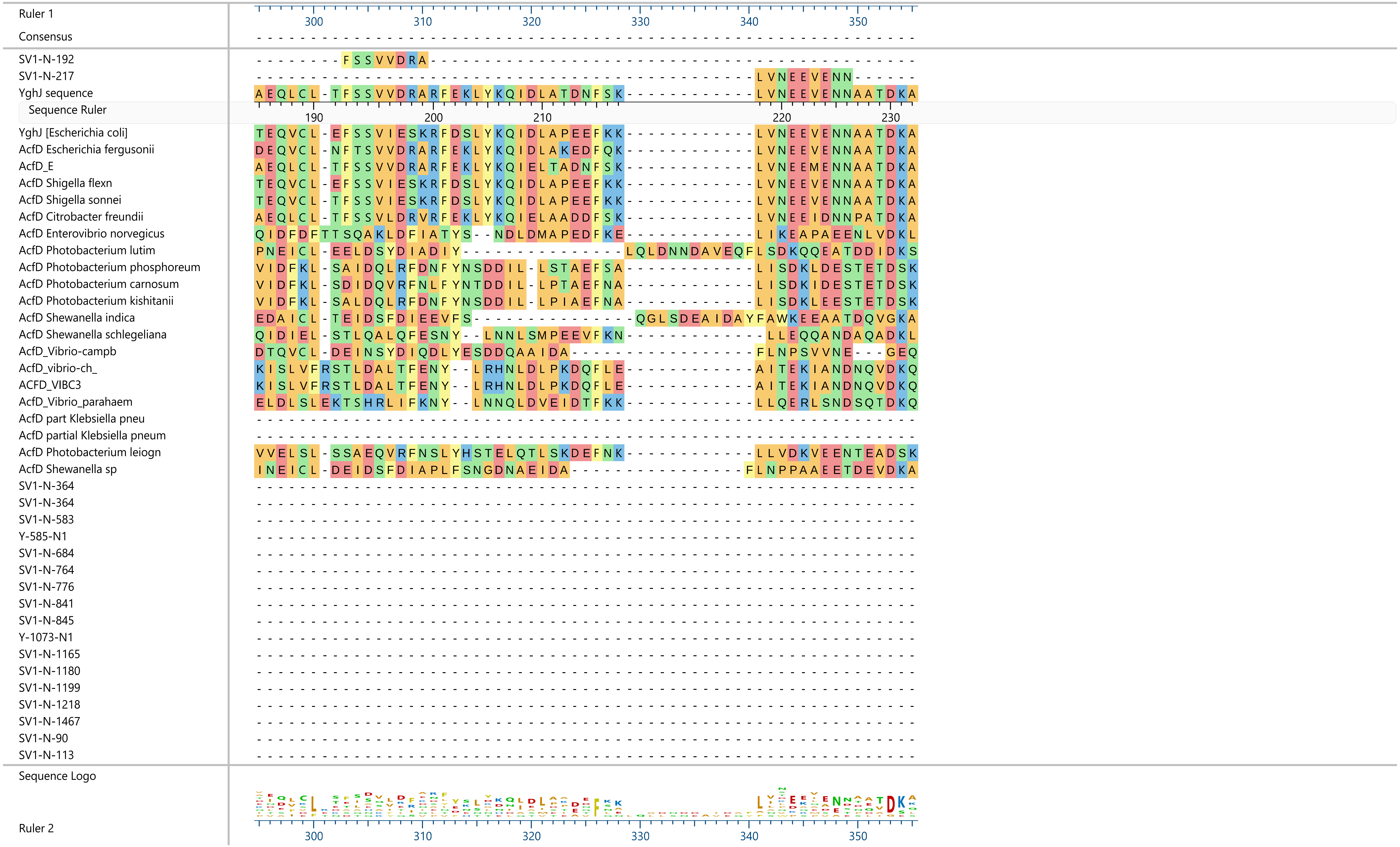

Ruler 1
300
310
320
330
340
350
Consensus
SV1-N-192
SV1-N-217
YghJ sequence
Sequence Ruler
190
200
210
220
230
YghJ [Escherichia coli]
AcfD Escherichia fergusonii
AcfD_E
AcfD Shigella flexn
AcfD Shigella sonnei
AcfD Citrobacter freundii
AcfD Enterovibrio norvegicus
AcfD Photobacterium lutim
AcfD Photobacterium phosphoreum
AcfD Photobacterium carnosum
AcfD Photobacterium kishitanii
AcfD Shewanella indica
AcfD Shewanella schlegeliana
AcfD_Vibrio-campb
AcfD_vibrio-ch_
ACFD_VIBC3
AcfD_Vibrio_parahaem
AcfD part Klebsiella pneu
AcfD partial Klebsiella pneum
AcfD Photobacterium leiogn
AcfD Shewanella sp
SV1-N-364
SV1-N-364
SV1-N-583
Y-585-N1
SV1-N-684
SV1-N-764
SV1-N-776
SV1-N-841
SV1-N-845
Y-1073-N1
SV1-N-1165
SV1-N-1180
SV1-N-1199
SV1-N-1218
SV1-N-1467
SV1-N-90
SV1-N-113
Sequence Logo
Ruler 2
300
310
320
330
340
350

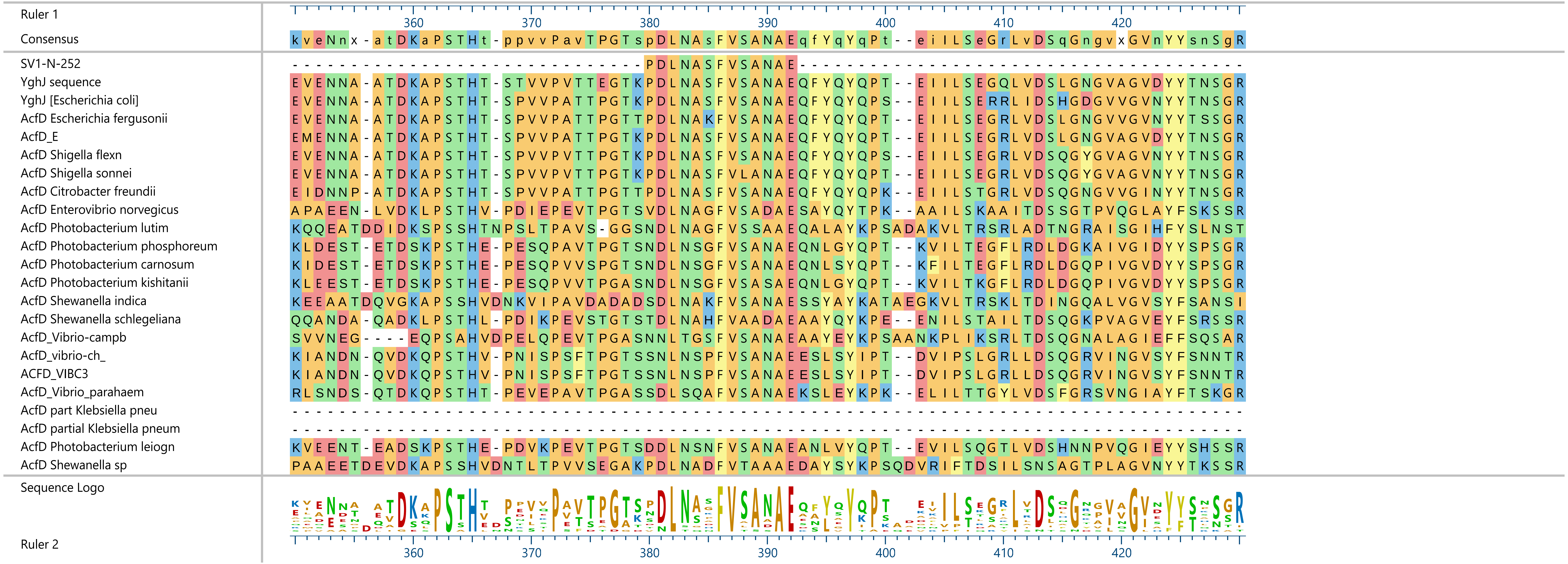
Ruler 1
Consensus
SV1-N-252
YghJ sequence
YghJ [Escherichia coli]
AcfD Escherichia fergusonii
AcfD_E
AcfD Shigella flexn
AcfD Shigella sonnei
AcfD Citrobacter freundii
AcfD Enterovibrio norvegicus
AcfD Photobacterium lutim
AcfD Photobacterium phosphoreum
AcfD Photobacterium carnosum
AcfD Photobacterium kishitanii
AcfD Shewanella indica
AcfD Shewanella schlegeliana
AcfD_Vibrio-campb
AcfD_vibrio-ch_
ACFD_VIBC3
AcfD_Vibrio_parahaem
AcfD part Klebsiella pneu
AcfD partial Klebsiella pneum
AcfD Photobacterium leiogn
AcfD Shewanella sp
Sequence Logo
Ruler 2
360
370
380
390
400
410
420

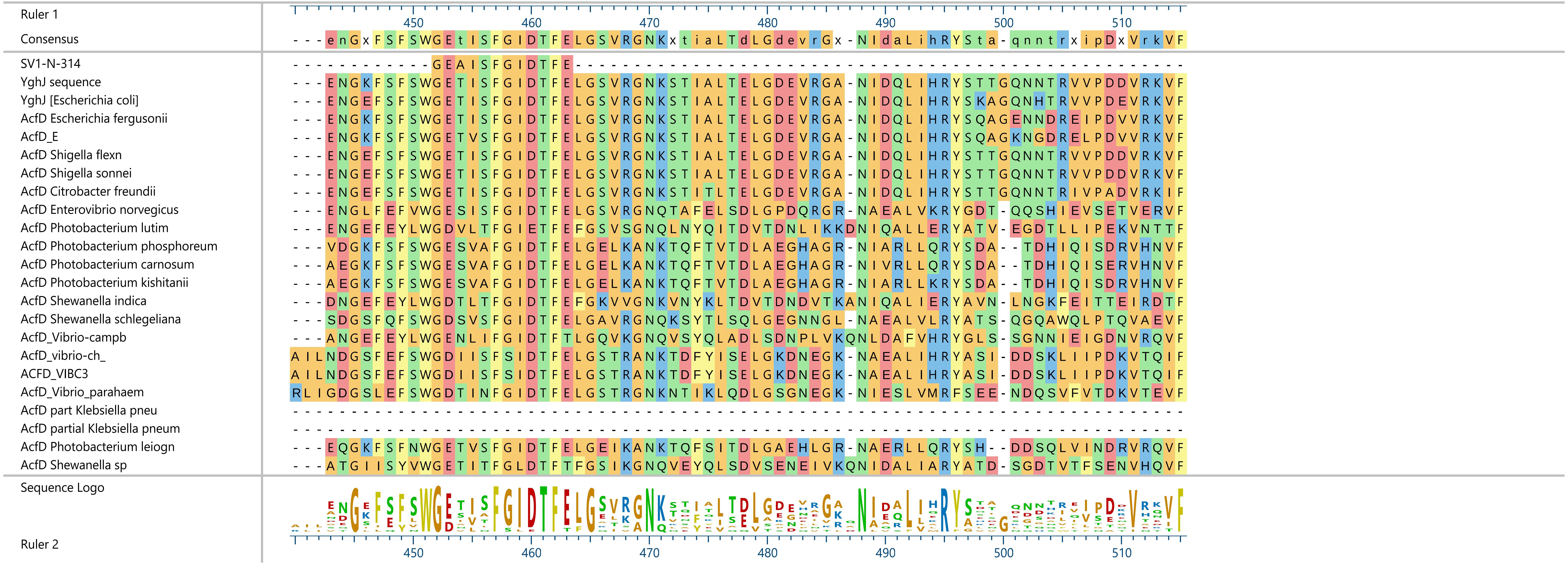
Ruler 1
450 460 470 480 490 500 510
Consensus ---enGxFSFSWGEtISFGIDTFELGSVRGNKxtiaLTdLGdevrGx-NIdaLihRYSta-qnntrxipDxVrkVF
SV1-N-314 ------------GEAISFGIDTFE
YghJ sequence ---ENGKFSFSWGETISFGIDTFELGSVRGNKSTIALTELGDEVRGA-NIDQLIHRYSTTGQNNTRVVPDDVRKVF
YghJ [Escherichia coli] ---ENGEFSFSWGETISFGIDTFELGSVRGNKSTIALTELGDEVRGA-NIDQLIHRYSKAGQNHTRVVPDEVRKVF
AcfD Escherichia fergusonii ---ENGKFSFSWGETISFGIDTFELGSVRGNKSTIALTELGDEVRGA-NIDQLIHRYSQAGENNDREIPDVVRKVF
AcfD_E ---ENGKFSFSWGETVSFGIDTFELGSVRGNKSTIALTELGDEVRGA-NIDQLIHRYSQAGKNGDRELPDVVRKVF
AcfD Shigella flexn ---ENGEFSFSWGETISFGIDTFELGSVRGNKSTIALTELGDEVRGA-NIDQLIHRYSTTGQNNTRVVPDDVRKVF
AcfD Shigella sonnei ---ENGEFSFSWGETISFGIDTFELGSVRGNKSTIALTELGDEVRGA-NIDQLIHRYSTTGQNNTRVVPDDVRKVF
AcfD Citrobacter freundii ---ENGEFSFSWGETISFGIDTFELGSVRGNKSTITLTELGDEVRGA-NIDQLIHRYSTTGQNNTRIVPADVRKIF
AcfD Enterovibrio norvegicus ---ENGLFEFVWGESISFGIDTFELGSVRGNQTAFELSDLGPDQRGR-NAEALVKRYGDT-QQSHIEVSETVERVF
AcfD Photobacterium lutim ---ENGEFEYLWGDVLTFGIETFEFGSVSGNQLNYQITDVTDNLIKKDNIQALLERYATV-EGDTLLIPEKVNTTF
AcfD Photobacterium phosphoreum ---VDGKFSFSWGESVAFGIDTFELGELKANKTQFTVTDLAEGHAGR-NIARLLQRYSDA--TDHIQISDRVHNVF
AcfD Photobacterium carnosum ---AEGKFSFSWGESVAFGIDTFELGELKANKTQFTVTDLAEGHAGR-NIVRLLQRYSDA--TDHIQISERVHNVF
AcfD Photobacterium kishitanii ---AEGKFSFSWGESVAFGIDTFELGELKANKTQFTVTDLAEGHAGR-NIARLLKRYSDA--TDHIQISDRVHNVF
AcfD Shewanella indica ---DNGEFEYLWGDTLTFGIDTFEFGKVVGNKVNYKLTDVTDNDVTKANIQALIERYAVN-LNGKFEITTEIRDTF
AcfD Shewanella schlegeliana ---SDGSFQFSWGDSVSFGIDTFELGAVRGNQKSYTLSQLGEGNNGL-NAEALVLRYATS-QGQAWQLPTQVAEVF
AcfD_Vibrio-campb ---ANGEFEYLWGENLIFGIDTFTLGQVKGNQVSYQLADLSDNPLVKQNLDAFVHRYGLS-SGNNIEIGDNVRQVF
AcfD_vibrio-ch_ AILNDGSFEFSWGDIISFSIDTFELGSTRANKTDFYISELGKDNEGK-NAEALIHRYASI-DDSKLIIPDKVTQIF
ACFD_VIBC3 AILNDGSFEFSWGDIISFSIDTFELGSTRANKTDFYISELGKDNEGK-NAEALIHRYASI-DDSKLIIPDKVTQIF
AcfD_Vibrio_parahaem RLIGDGSLEFSWGDTINFGIDTFELGSTRGNKNTIKLQDLGSGNEGK-NIESLVMRFSEE-NDQSVFVTDKVTEVF
AcfD part Klebsiella pneu
AcfD partial Klebsiella pneum
AcfD Photobacterium leiogn ---EQGKFSFNWGETVSFGIDTFELGEIKANKTQFSITDLGAEHLGR-NAERLLQRYSH--DDSQLVINDRVRQVF
AcfD Shewanella sp ---ATGIISYVWGETITFGLDTFTFGSIKGNQVEYQLSDVSENEIVKQNIDALIARYATD-SGDTVTFSENVHQVF
Sequence Logo
Ruler 2
450 460 470 480 490 500 510

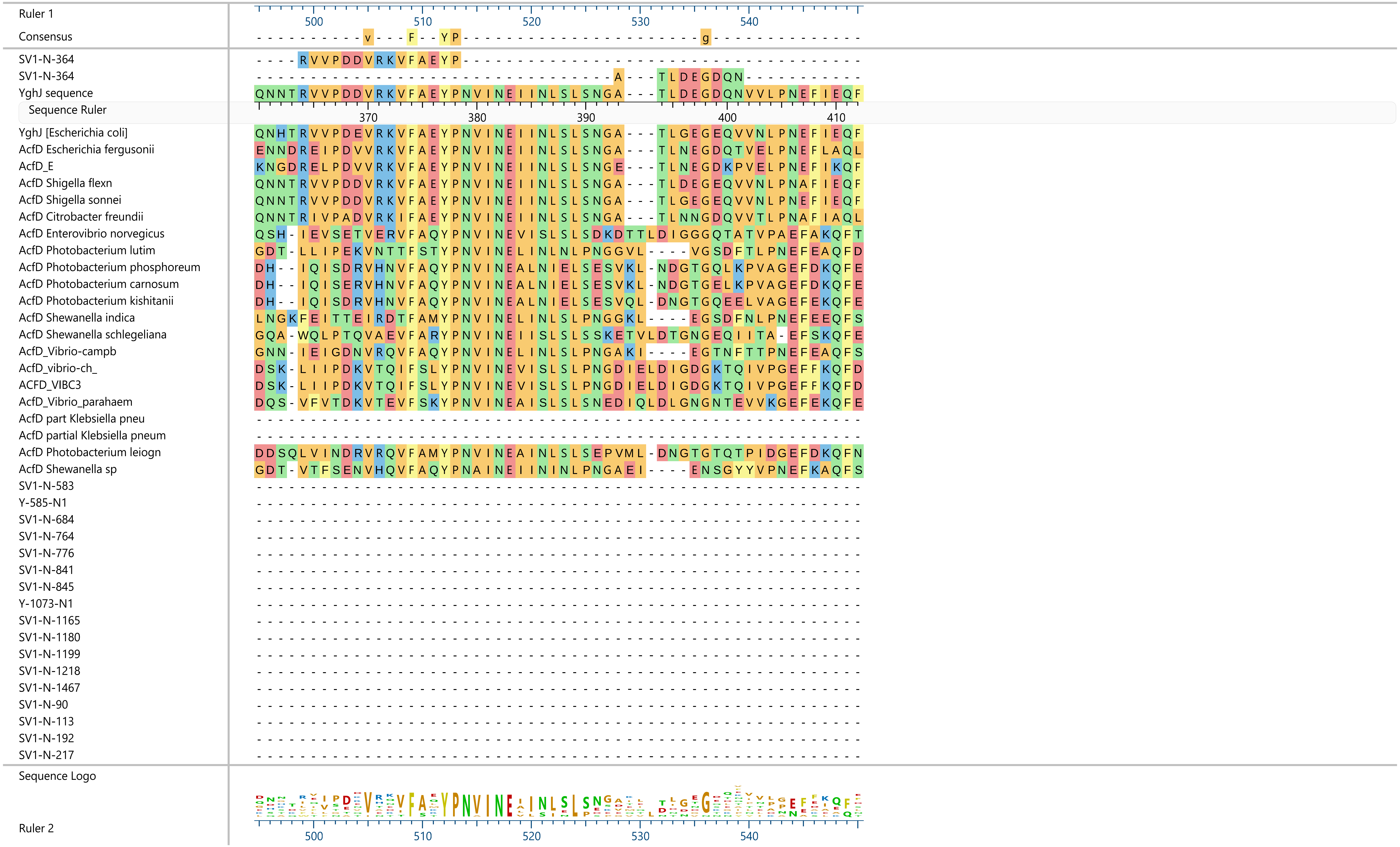

Ruler 1
500 510 520 530 540
Consensus
v F YP g
SV1-N-364 ----RVVPDDVRKVFAEYP
SV1-N-364 A---TLDEGDQN
YghJ sequence QNNTRVVPDDVRKVFAEYPNVINEIINLSLSNGA---TLDEGDQNVVLPNEFIEQF
Sequence Ruler
370 380 390 400 410
YghJ [Escherichia coli] QNHTRVVPDEVRKVFAEYPNVINEIINLSLSNGA---TLGEGEQVVNLPNEFIEQF
AcfD Escherichia fergusonii ENNDREIPDVVRKVFAEYPNVINEIINLSLSNGA---TLNEGDQTVELPNEFLAQL
AcfD_E KNGDRELPDVVRKVFAEYPNVINEIINLSLSNGE---TLNEGDKPVELPNEFIKQF
AcfD Shigella flexn QNNTRVVPDDVRKVFAEYPNVINEIINLSLSNGA---TLDEGEQVVNLPNAFIEQF
AcfD Shigella sonnei QNNTRVVPDDVRKVFAEYPNVINEIINLSLSNGA---TLGEGEQVVNLPNEFIEQF
AcfD Citrobacter freundii QNNTRIVPADVRKIFAEYPNVINEIINLSLSNGA---TLNNGDQVVTLPNAFIAQL
AcfD Enterovibrio norvegicus QSH-IEVSETVERVFAQYPNVINEVISLSLSDKDTTLDIGGGQTATVPAEFAKQFT
AcfD Photobacterium lutim GDT-LLIPEKVNTTFSTYPNVINELINLNLPNGGVL----VGSDFTLPNEFEAQFD
AcfD Photobacterium phosphoreum DH--IQISDRVHNVFAQYPNVINEALNIELSESVKL-NDGTGQLKPVAGEFDKQFE
AcfD Photobacterium carnosum DH--IQISERVHNVFAQYPNVINEALNIELSESVKL-NDGTGELKPVAGEFDKQFE
AcfD Photobacterium kishitanii DH--IQISDRVHNVFAQYPNVINEALNIELSESVQL-DNGTGQEELVAGEFEKQFE
AcfD Shewanella indica LNGKFEITTEIRDTFAMYPNVINELINLSLPNGGKL----EGSDFNLPNEFEEQFS
AcfD Shewanella schlegeliana GQA-WQLPTQVAEVFARYPNVINEIISLSLSSKETVLDTGNGEQIITA-EFSKQFE
AcfD_Vibrio-campb GNN-IEIGDNVRQVFAQYPNVINELINLSLPNGAKI----EGTNFTTPNEFEAQFS
AcfD_vibrio-ch_ DSK-LIIPDKVTQIFSLYPNVINEVISLSLPNGDIELDIGDGKTQIVPGEFFKQFD
ACFD_VIBC3 DSK-LIIPDKVTQIFSLYPNVINEVISLSLPNGDIELDIGDGKTQIVPGEFFKQFD
AcfD_Vibrio_parahaem DQS-VFVTDKVTEVFSKYPNVINEAISLSLSNEDIQLDLGNGNTEVVKGEFEKQFE
AcfD part Klebsiella pneu
AcfD partial Klebsiella pneum
AcfD Photobacterium leiogn DDSQLVINDRVRQVFAMYPNVINEAINLSLSEPVML-DNGTGTQTPIDGEFDKQFN
AcfD Shewanella sp GDT-VTFSENVHQVFAQYPNAINEIININLPNGAEI----ENSGYYVPNEFKAQFS
SV1-N-583
Y-585-N1
SV1-N-684
SV1-N-764
SV1-N-776
SV1-N-841
SV1-N-845
Y-1073-N1
SV1-N-1165
SV1-N-1180
SV1-N-1199
SV1-N-1218
SV1-N-1467
SV1-N-90
SV1-N-113
SV1-N-192
SV1-N-217
Sequence Logo
Ruler 2
500 510 520 530 540

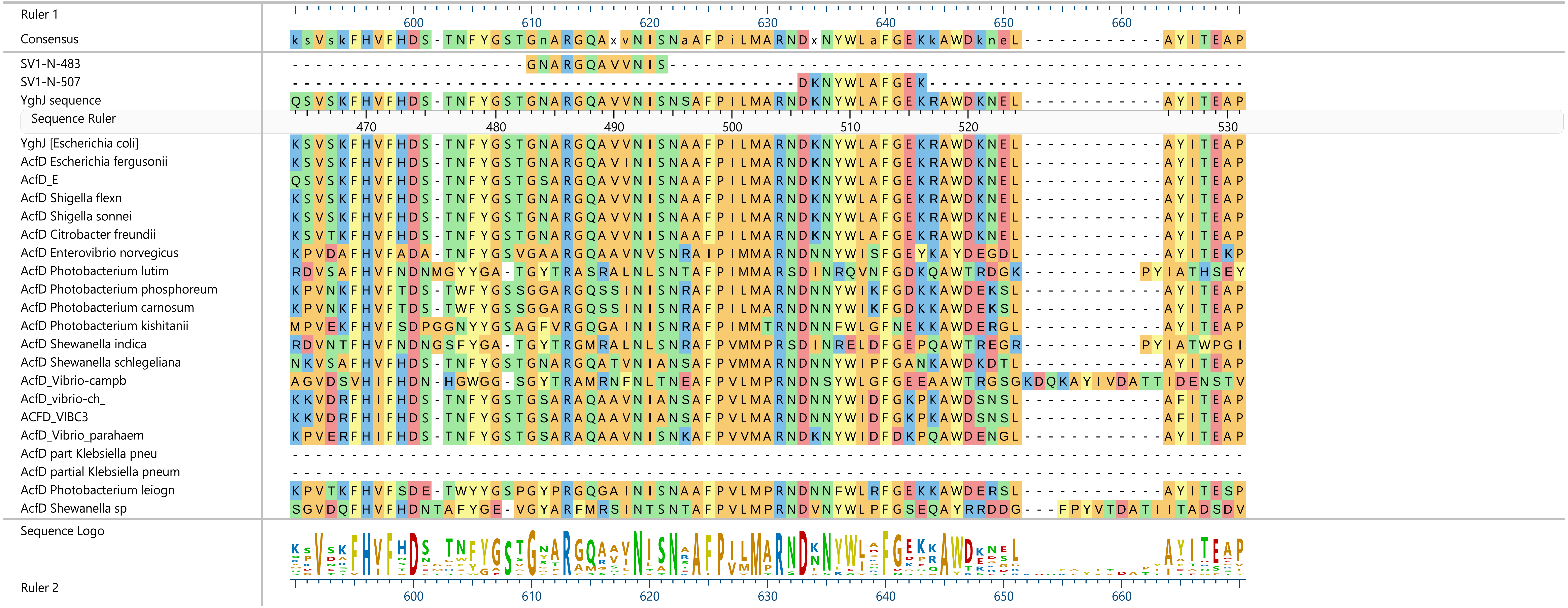

Ruler 1
600 610 620 630 640 650 660
Consensus
ksVskFHVFHDS-TNFYGSTGnARGQAxvNISNaAFPiLMARNDxNYWLaFGEKkAWDkneL------------AYITEAP
SV1-N-483
--------------------GNARGQAVVNIS---
SV1-N-507
---DKNYWLAFGEK---------------------------
YghJ sequence
QSVSKFHVFHDS-TNFYGSTGNARGQAVVNISNSAFPILMARNDKNYWLAFGEKRAWDKNEL------------AYITEAP
Sequence Ruler
470 480 490 500 510 520 530
YghJ [Escherichia coli]
KSVSKFHVFHDS-TNFYGSTGNARGQAVVNISNAAFPILMARNDKNYWLAFGEKRAWDKNEL------------AYITEAP
AcfD Escherichia fergusonii
KSVSKFHVFHDS-TNFYGSTGNARGQAVINISNAAFPILMARNDKNYWLAFGEKRAWDKNEL------------AYITEAP
AcfD_E
QSVSKFHVFHDS-TNFYGSTGSARGQAVVNISNAAFPILMARNDKNYWLAFGEKRAWDKNEL------------AYITEAP
AcfD Shigella flexn
KSVSKFHVFHDS-TNFYGSTGNARGQAVVNISNAAFPILMARNDKNYWLAFGEKRAWDKNEL------------AYITEAP
AcfD Shigella sonnei
KSVSKFHVFHDS-TNFYGSTGNARGQAVVNISNAAFPILMARNDKNYWLAFGEKRAWDKNEL------------AYITEAP
AcfD Citrobacter freundii
KSVTKFHVFHDS-TNFYGSTGNARGQAVVNISNAAFPILMARNDKNYWLAFGEKRAWDKNEL------------AYITEAP
AcfD Enterovibrio norvegicus
KPVDAFHVFADA-TNFYGSVGAARGQAAVNVSNRAIPIMMARNDNNYWISFGEYKAYDEGDL------------AYITEKP
AcfD Photobacterium lutim
RDVSAFHVFNDNMGYYGA-TGYTRASRALNLSNTAFPIMMARSDINRQVNFGDKQAWTRDGK----------PYIATHSEY
AcfD Photobacterium phosphoreum
KPVNKFHVFTDS-TWFYGSSGGARGQSSINISNRAFPILMARNDNNYWIKFGDKKAWDEKSL------------AYITEAP
AcfD Photobacterium carnosum
KPVNKFHVFTDS-TWFYGSSGGARGQSSINISNRAFPILMARNDNNYWIKFGDKKAWDEKSL------------AYITEAP
AcfD Photobacterium kishitanii
MPVEKFHVFSDPGGNYYGSAGFVRGQGAINISNRAFPIMMTRNDNNFWLGFNEKKAWDERGL------------AYITEAP
AcfD Shewanella indica
RDVNTFHVFNDNGSFYGA-TGYTRGMRALNLSNRAFPVMMPRSDINRELDFGEPQAWTREGR----------PYIATWPGI
AcfD Shewanella schlegeliana
NKVSAFHVFHDS-TNFYGSTGNARGQATVNIANSAFPVMMARNDNNYWIPFGANKAWDKDTL------------AYITEAP
AcfD_Vibrio-campb
AGVDSVHIFHDN-HGWGG-SGYTRAMRNFNLTNEAFPVLMPRNDNSYWLGFGEEAAWTRGSGKDQKAYIVDATTIDENSTV
AcfD_vibrio-ch_
KKVDRFHIFHDS-TNFYGSTGSARAQAAVNIANSAFPVLMARNDNNYWIDFGKPKAWDSNSL------------AFITEAP
ACFD_VIBC3
KKVDRFHIFHDS-TNFYGSTGSARAQAAVNIANSAFPVLMARNDNNYWIDFGKPKAWDSNSL------------AFITEAP
AcfD_Vibrio_parahaem
KPVERFHIFHDS-TNFYGSTGSARAQAAVNISNKAFPVVMARNDKNYWIDFDKPQAWDENGL------------AYITEAP
AcfD part Klebsiella pneu

AcfD partial Klebsiella pneum

AcfD Photobacterium leiogn
KPVTKFHVFSDE-TWYYGSPGYPRGQGAINISNAAFPVLMPRNDNNFWLRFGEKKAWDERSL------------AYITESP
AcfD Shewanella sp
SGVDQFHVFHDNTAFYGE-VGYARFMRSINTSNTAFPVLMPRNDVNYWLPFGSEQAYRRDDG---FPYVTDATIITADSDV
Sequence Logo
Ruler 2
600 610 620 630 640 650 660

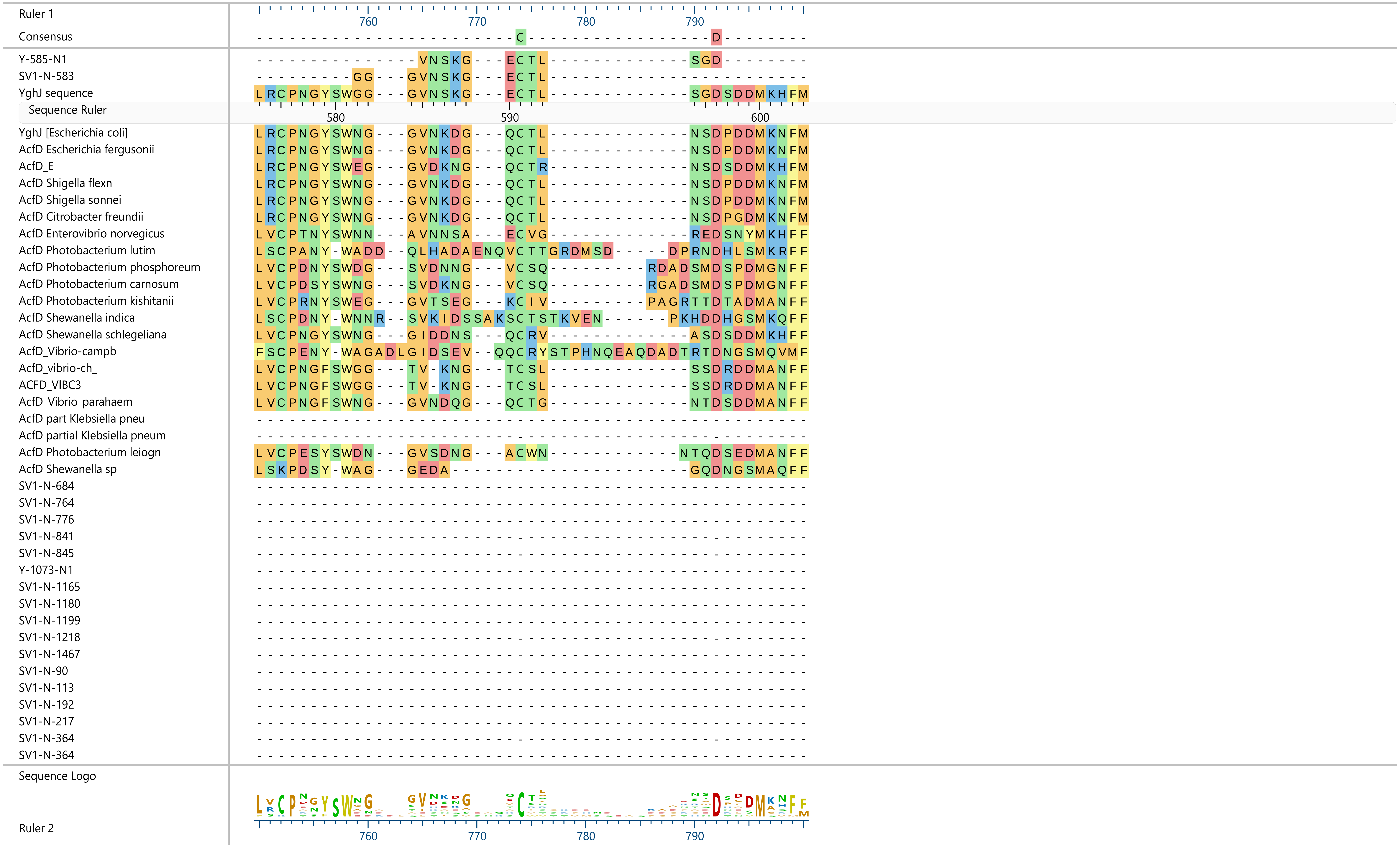
Ruler 1
760 770 780 790
Consensus ------------------------C-----------------D--------
Y-585-N1 ---------------VNSKG---ECTL-------------SGD--------
SV1-N-583 ---------GG---GVNSKG---ECTL------------------------
YghJ sequence LRCPNGYSWGG---GVNSKG---ECTL-------------SGDSDDMKHFM
Sequence Ruler
580 590 600
YghJ [Escherichia coli] LRCPNGYSWNG---GVNKDG---QCTL-------------NSDPDDMKNFM
AcfD Escherichia fergusonii LRCPNGYSWNG---GVNKDG---QCTL-------------NSDPDDMKNFM
AcfD_E LRCPNGYSWEG---GVDKNG---QCTR-------------NSDSDDMKHFM
AcfD Shigella flexn LRCPNGYSWNG---GVNKDG---QCTL-------------NSDPDDMKNFM
AcfD Shigella sonnei LRCPNGYSWNG---GVNKDG---QCTL-------------NSDPDDMKNFM
AcfD Citrobacter freundii LRCPNGYSWNG---GVNKDG---QCTL-------------NSDPGDMKNFM
AcfD Enterovibrio norvegicus LVCPTNYSWNN---AVNNSA---ECVG-------------REDSNYMKHFF
AcfD Photobacterium lutim LSCPANY-WADD--QLHADAENQVCTTGRDMSD-----DPRNDHLSMKRFF
AcfD Photobacterium phosphoreum LVCPDNYSWDG---SVDNNG---VCSQ---------RDADSMDSPDMGNFF
AcfD Photobacterium carnosum LVCPDSYSWNG---SVDKNG---VCSQ---------RGADSMDSPDMGNFF
AcfD Photobacterium kishitanii LVCPRNYSWEG---GVTSEG---KCIV---------PAGRTTDTADMANFF
AcfD Shewanella indica LSCPDNY-WNNR--SVKIDSSAKSCTSTKVEN------PKHDDHGSMKQFF
AcfD Shewanella schlegeliana LVCPNGYSWNG---GIDDNS---QCRV-------------ASDSDDMKHFF
AcfD_Vibrio-campb FSCPENY-WAGADLGIDSEV--QQCRYSTPHNQEAQDADTRTDNGSMQVMF
AcfD_vibrio-ch_ LVCPNGFSWGG---TV-KNG---TCSL-------------SSDRDDMANFF
ACFD_VIBC3 LVCPNGFSWGG---TV-KNG---TCSL-------------SSDRDDMANFF
AcfD_Vibrio_parahaem LVCPNGFSWNG---GVNDQG---QCTG-------------NTDSDDMANFF
AcfD part Klebsiella pneu ---
AcfD partial Klebsiella pneum ---
AcfD Photobacterium leiogn LVCPESYSWDN---GVSDNG---ACWN------------NTQDSEDMANFF
AcfD Shewanella sp LSKPDSY-WAG---GEDA----------------------GQDNGSMAQFF
SV1-N-684 ---
SV1-N-764 ---
SV1-N-776 ---
SV1-N-841 ---
SV1-N-845 ---
Y-1073-N1 ---
SV1-N-1165 ---
SV1-N-1180 ---
SV1-N-1199 ---
SV1-N-1218 ---
SV1-N-1467 ---
SV1-N-90 ---
SV1-N-113 ---
SV1-N-192 ---
SV1-N-217 ---
SV1-N-364 ---
SV1-N-364 ---
Sequence Logo
Ruler 2
760 770 780 790

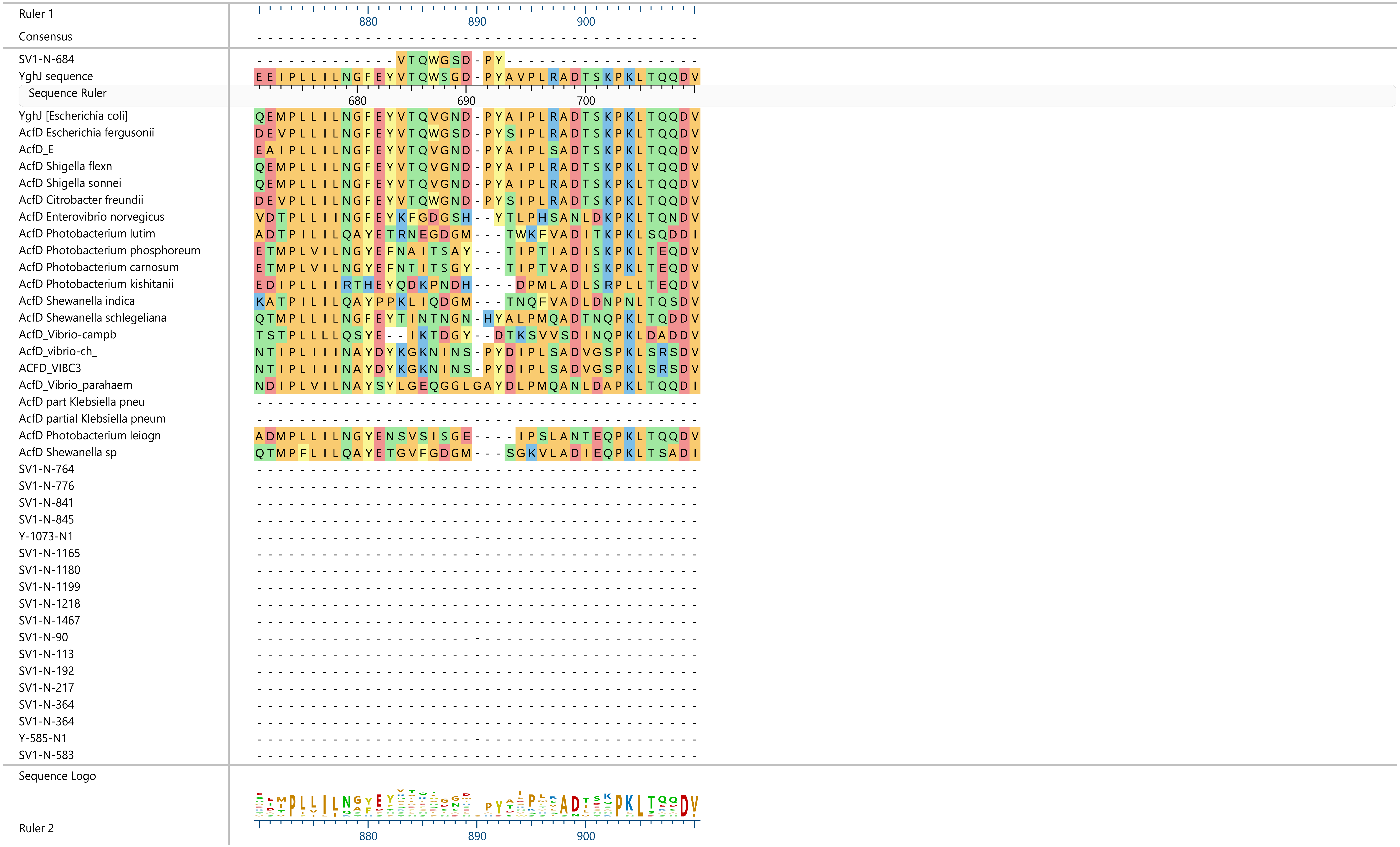
Ruler 1
880
890
900
Consensus
SV1-N-684 -------------VTQWGSD-PY------------------
YghJ sequence EEIPLLILNGFEYVTQWSGD-PYAVPLRADTSKPKLTQQDV
Sequence Ruler
680
690
700
YghJ [Escherichia coli] QEMPLLILNGFEYVTQVGND-PYAIPLRADTSKPKLTQQDV
AcfD Escherichia fergusonii DEVPLLILNGFEYVTQWGSD-PYSIPLRADTSKPKLTQQDV
AcfD_E EAIPLLILNGFEYVTQVGND-PYAIPLSADTSKPKLTQQDV
AcfD Shigella flexn QEMPLLILNGFEYVTQVGND-PYAIPLRADTSKPKLTQQDV
AcfD Shigella sonnei QEMPLLILNGFEYVTQVGND-PYAIPLRADTSKPKLTQQDV
AcfD Citrobacter freundii DEVPLLILNGFEYVTQWGND-PYSIPLRADTSKPKLTQQDV
AcfD Enterovibrio norvegicus VDTPLLIINGFEYKFGDGSH--YTLPHSANLDKPKLTQNDV
AcfD Photobacterium lutim ADTPILILQAYETRNEGDGM---TWKFVADITKPKLSQDDI
AcfD Photobacterium phosphoreum ETMPLVILNGYEFNAITSAY---TIPTIADISKPKLTEQDV
AcfD Photobacterium carnosum ETMPLVILNGYEFNTITSGY---TIPTVADISKPKLTEQDV
AcfD Photobacterium kishitanii EDIPLLIIRTHEYQDKPNDH----DPMLADLSRPLLTEQDV
AcfD Shewanella indica KATPILILQAYPPKLIQDGM---TNQFVADLDNPNLTQSDV
AcfD Shewanella schlegeliana QTMPLLILNGFEYTINTNGN-HYALPMQADTNQPKLTQDDV
AcfD_Vibrio-campb TSTPLLLQSYE--IKTDGY--DTKSVVSDINQPKLDADDV
AcfD_vibrio-ch_ NTIPLIIINAYDYKGKNINS-PYDIPLSADVGSPKLSRSDV
ACFD_VIBC3 NTIPLIIINAYDYKGKNINS-PYDIPLSADVGSPKLSRSDV
AcfD_Vibrio_parahaem NDIPLVILNAYSYLGEQGGLGAYDLPMQANLDAPKLTQQDI
AcfD part Klebsiella pneu
AcfD partial Klebsiella pneum
AcfD Photobacterium leiogn ADMPLLILNGYENSVSISGE----IPSLANTEQPKLTQQDV
AcfD Shewanella sp QTMPFLILQAYETGVFGDGM---SGKVLADIEQPKLTSADI
SV1-N-764
SV1-N-776
SV1-N-841
SV1-N-845
Y-1073-N1
SV1-N-1165
SV1-N-1180
SV1-N-1199
SV1-N-1218
SV1-N-1467
SV1-N-90
SV1-N-113
SV1-N-192
SV1-N-217
SV1-N-364
SV1-N-364
Y-585-N1
SV1-N-583
Sequence Logo
Ruler 2
880
890
900

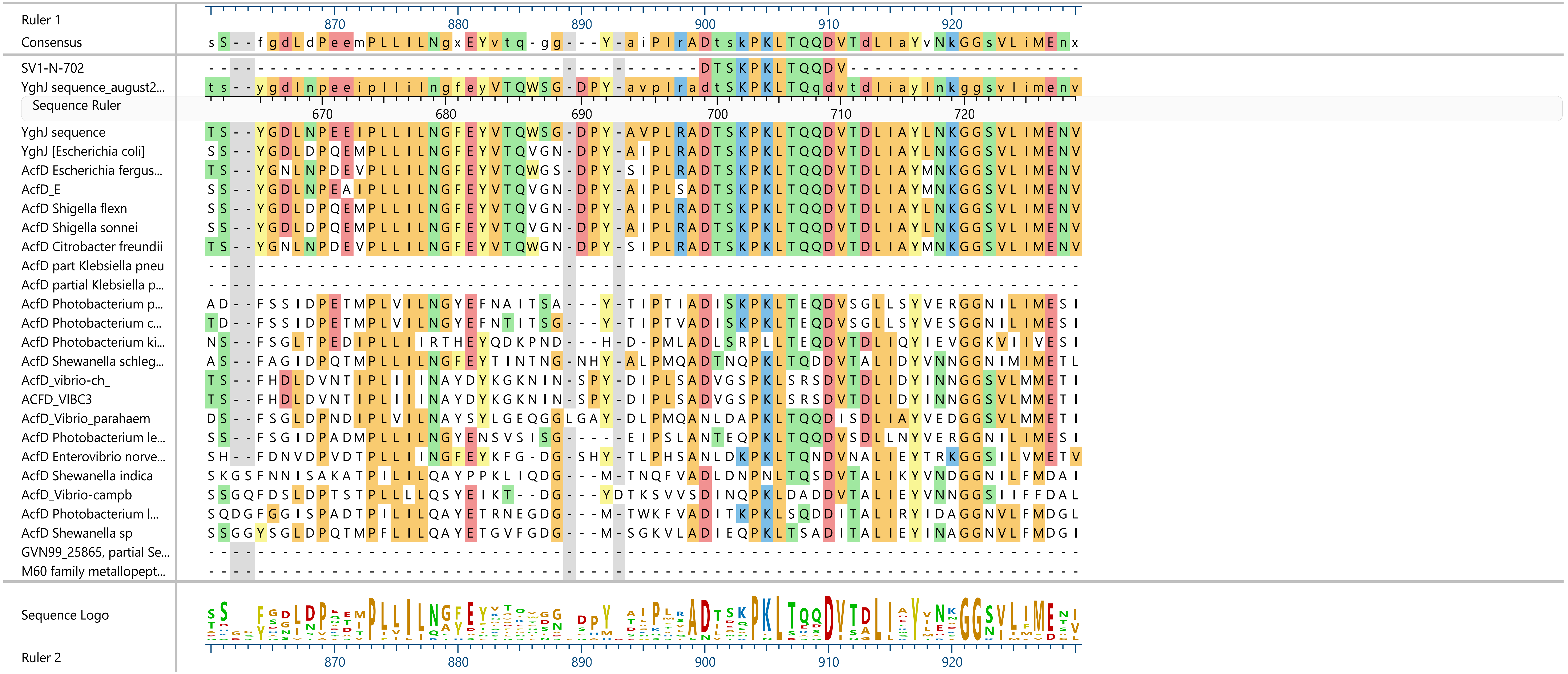
Ruler 1
Consensus
SV1-N-702
YghJ sequence_august2...
Sequence Ruler
YghJ sequence
YghJ [Escherichia coli]
AcfD Escherichia fergus...
AcfD_E
AcfD Shigella flexn
AcfD Shigella sonnei
AcfD Citrobacter freundii
AcfD part Klebsiella pneu
AcfD partial Klebsiella p...
AcfD Photobacterium p...
AcfD Photobacterium c...
AcfD Photobacterium ki...
AcfD Shewanella schleg...
AcfD_vibrio-ch_
ACFD_VIBC3
AcfD_Vibrio_parahaem
AcfD Photobacterium le...
AcfD Enterovibrio norve...
AcfD Shewanella indica
AcfD_Vibrio-campb
AcfD Photobacterium l...
AcfD Shewanella sp
GVN99_25865, partial Se...
M60 family metallopept...
Sequence Logo
Ruler 2

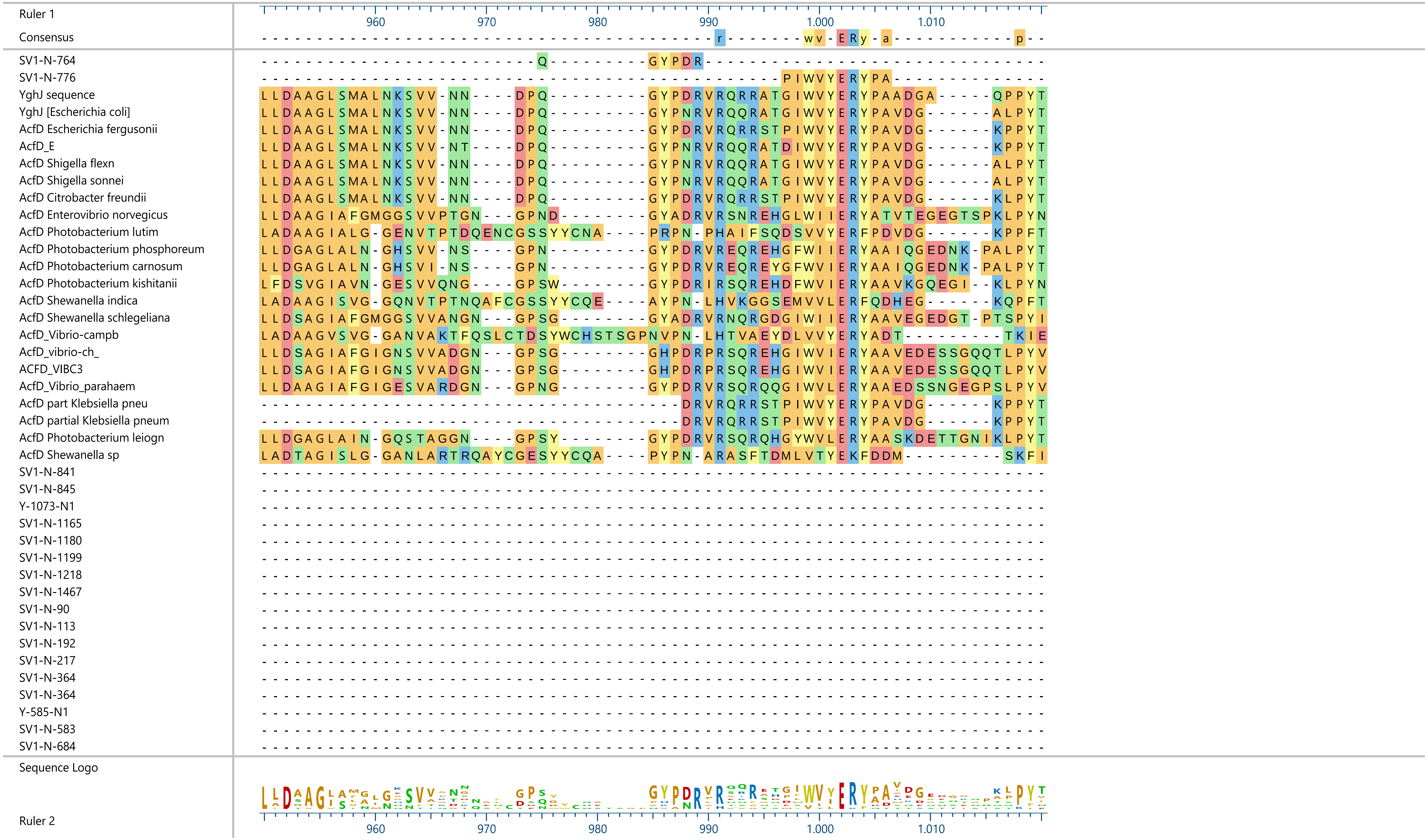
Ruler 1
Consensus
SV1-N-764
SV1-N-776
YghJ sequence
YghJ [Escherichia coli]
AcfD Escherichia fergusonii
AcfD_E
AcfD Shigella flexn
AcfD Shigella sonnei
AcfD Citrobacter freundii
AcfD Enterovibrio norvegicus
AcfD Photobacterium lutim
AcfD Photobacterium phosphoreum
AcfD Photobacterium carnosum
AcfD Photobacterium kishitanii
AcfD Shewanella indica
AcfD Shewanella schlegeliana
AcfD_Vibrio-campb
AcfD_vibrio-ch_
ACFD_VIBC3
AcfD_Vibrio_parahaem
AcfD part Klebsiella pneu
AcfD partial Klebsiella pneum
AcfD Photobacterium leiogn
AcfD Shewanella sp
SV1-N-841
SV1-N-845
Y-1073-N1
SV1-N-1165
SV1-N-1180
SV1-N-1199
SV1-N-1218
SV1-N-1467
SV1-N-90
SV1-N-113
SV1-N-192
SV1-N-217
SV1-N-364
SV1-N-364
Y-585-N1
SV1-N-583
SV1-N-684
Sequence Logo
Ruler 2
960
970
980
990
1.000
1.010

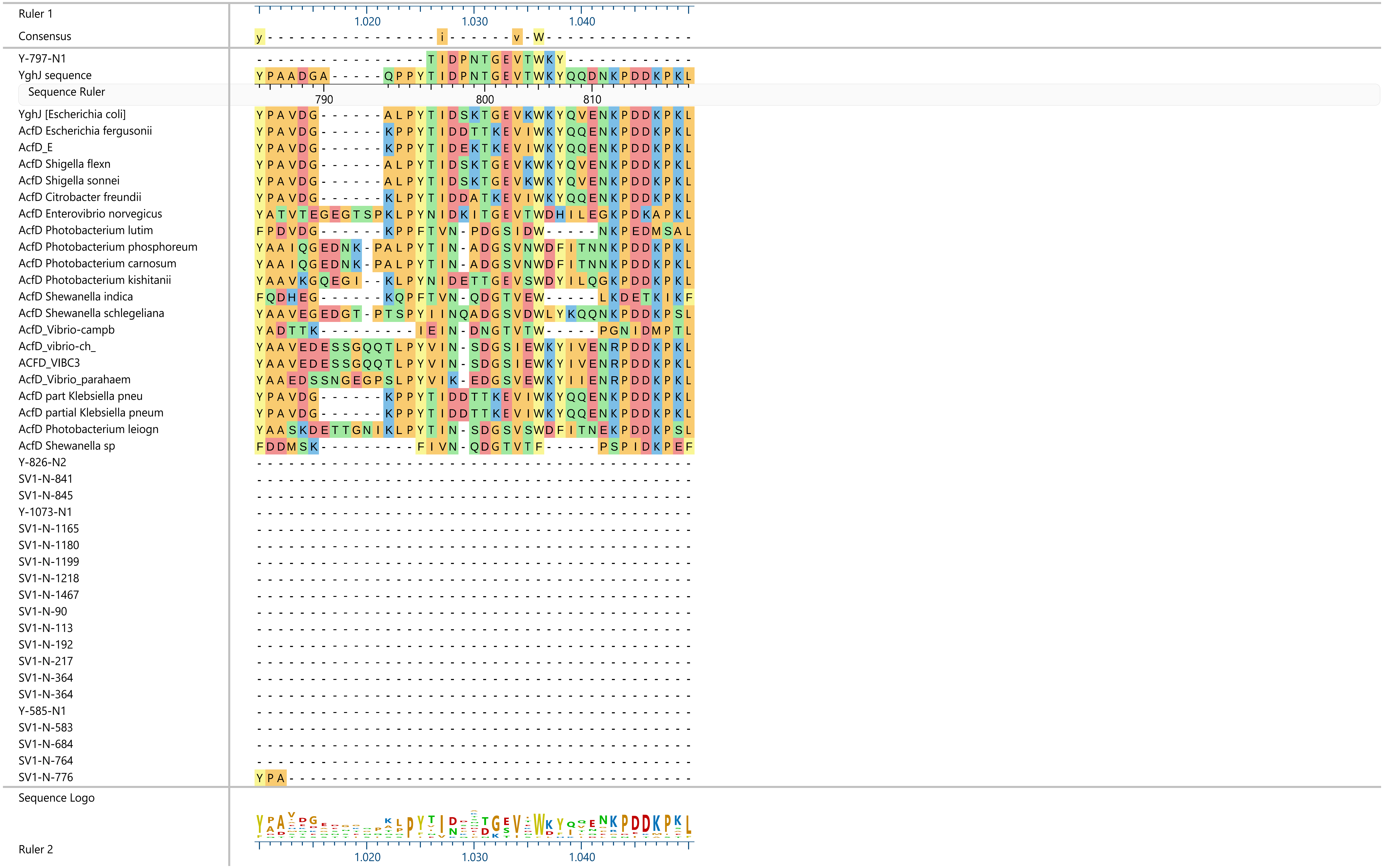

Ruler 1
1.020
1.030
1.040
Consensus y----------------i------v-W--------------
Y-797-N1 ----------------TIDPNTGEVTWKY------------
YghJ sequence YPAADGA-----QPPYTIDPNTGEVTWKYQQDNKPDDKPKL
Sequence Ruler
790
800
810
YghJ [Escherichia coli] YPAVDG------ALPYTIDSKTGEVKWKYQVENKPDDKPKL
AcfD Escherichia fergusonii YPAVDG------KPPYTIDDTTKEVIWKYQQENKPDDKPKL
AcfD_E YPAVDG------KPPYTIDEKTKEVIWKYQQENKPDDKPKL
AcfD Shigella flexn YPAVDG------ALPYTIDSKTGEVKWKYQVENKPDDKPKL
AcfD Shigella sonnei YPAVDG------ALPYTIDSKTGEVKWKYQVENKPDDKPKL
AcfD Citrobacter freundii YPAVDG------KLPYTIDDATKEVIWKYQQENKPDDKPKL
AcfD Enterovibrio norvegicus YATVTEGEGTSPKLPYNIDKITGEVTWDHILEGKPDKAPKL
AcfD Photobacterium lutim FPDVDG------KPPFTVN-PDGSIDW-----NKPEDMSAL
AcfD Photobacterium phosphoreum YAAIQGEDNK-PALPYTIN-ADGSVNWDFITNNKPDDKPKL
AcfD Photobacterium carnosum YAAIQGEDNK-PALPYTIN-ADGSVNWDFITNNKPDDKPKL
AcfD Photobacterium kishitanii YAAVKGQEGI--KLPYNIDETTGEVSWDYILQGKPDDKPKL
AcfD Shewanella indica FQDHEG------KQPFTVN-QDGTVEW-----LKDETKIKF
AcfD Shewanella schlegeliana YAAVEGEDGT-PTSPYIINQADGSVDWLYKQQNKPDDKPSL
AcfD_Vibrio-campb YADTTK---------IEIN-DNGTVTW-----PGNIDMPTL
AcfD_vibrio-ch_ YAAVEDESSGQQTLPYVIN-SDGSIEWKYIVENRPDDKPKL
ACFD_VIBC3 YAAVEDESSGQQTLPYVIN-SDGSIEWKYIVENRPDDKPKL
AcfD_Vibrio_parahaem YAAEDSSNGEGPSLPYVIK-EDGSVEWKYIIENRPDDKPKL
AcfD part Klebsiella pneu YPAVDG------KPPYTIDDTTKEVIWKYQQENKPDDKPKL
AcfD partial Klebsiella pneum YPAVDG------KPPYTIDDTTKEVIWKYQQENKPDDKPKL
AcfD Photobacterium leiogn YAASKDETTGNIKLPYTIN-SDGSVSWDFITNEKPDDKPSL
AcfD Shewanella sp FDDMSK---------FIVN-QDGTVTF-----PSPIDKPEF
Y-826-N2 ---
SV1-N-841 ---
SV1-N-845 ---
Y-1073-N1 ---
SV1-N-1165 ---
SV1-N-1180 ---
SV1-N-1199 ---
SV1-N-1218 ---
SV1-N-1467 ---
SV1-N-90 ---
SV1-N-113 ---
SV1-N-192 ---
SV1-N-217 ---
SV1-N-364 ---
SV1-N-364 ---
Y-585-N1 ---
SV1-N-583 ---
SV1-N-684 ---
SV1-N-764 ---
SV1-N-776 YPA--------------------------------------
Sequence Logo
Ruler 2
1.020
1.030
1.040

	1.060 · 1.070 · 1.080 (Ruler 1)
Consensus	`-----------------------G--------Afi------`
Y-826-N2	`------------------QEEVEGK--QVTRY---------`
YghJ sequence	`LEVASW------------QEEVEGK--QVTRYAFIDEAEY-`
Sequence Ruler	820 · 830 · 840
YghJ [Escherichia coli]	`LEVASW------------LEDVDGK--QETCYAFIDEADH-`
AcfD Escherichia fergusonii	`LEVASW------------QEEVEGK--QVTQFAFIDEADH-`
AcfD_E	`LEVASW------------LEDVNGK--QETRYAFIDEAEH-`
AcfD Shigella flexn	`LEVASW------------LEDVDGK--QETRYAFIDEADH-`
AcfD Shigella sonnei	`LEVASW------------LEDVDGK--QETRYAFIDEADH-`
AcfD Citrobacter freundii	`LEVASW------------QEEVEGK--QVTQFAFIDEADH-`
AcfD Enterovibrio norvegicus	`LETASYAG----------FDH-EGK--PATLKAFIREEDHF`
AcfD Photobacterium lutim	`LEIPTY------------TDE-QGK----THYARIAF----`
AcfD Photobacterium phosphoreum	`LEVAKKTI----------TND-DGT--NVELLAFIDTYN--`
AcfD Photobacterium carnosum	`LEVAKKVI----------TND-EGK--NETVVAFIDTFN--`
AcfD Photobacterium kishitanii	`LEVARKSY----------FNMVTGK--HESQLAFIDTAG--`
AcfD Shewanella indica	`FEIPTYEVPKLNADGNPEFDP-QGNPVMLKKHARIFV----`
AcfD Shewanella schlegeliana	`LSVAKWVE----------TDE-DGV--ETTHTAFIDE----`
AcfD_Vibrio-campb	`LEIPLYKAS--------I----DGQ--EHQRYAFHMV----`
AcfD_vibrio-ch_	`LEVASWVE-----------SE-AGDK-LITHYAFIDESQHW`
ACFD_VIBC3	`LEVASWVE-----------SE-AGDK-LITHYAFIDESQHW`
AcfD_Vibrio_parahaem	`LEVAKWIE----------INE-QGD--SKVQVAFIDEANFY`
AcfD part Klebsiella pneu	`LEVASW------------QEEVEGK--QVTQFAFIDEADH-`
AcfD partial Klebsiella pneum	`LEVASW------------QEEVEGK--QVTQFAFIDEADH-`
AcfD Photobacterium leiogn	`LEVARKLI----------ENE-QGE--NVELLAFVDTYQEG`
AcfD Shewanella sp	`FGVAQYKT----------IAA-DGI--EHNNFAFFSA----`
Y-1073-N1	`-----------------------------------------`
SV1-N-1165	`-----------------------------------------`
SV1-N-1180	`-----------------------------------------`
SV1-N-1199	`-----------------------------------------`
SV1-N-1218	`-----------------------------------------`
SV1-N-1467	`-----------------------------------------`
SV1-N-90	`-----------------------------------------`
SV1-N-113	`-----------------------------------------`
SV1-N-192	`-----------------------------------------`
SV1-N-217	`-----------------------------------------`
SV1-N-364	`-----------------------------------------`
SV1-N-364	`-----------------------------------------`
Y-585-N1	`-----------------------------------------`
SV1-N-583	`-----------------------------------------`
SV1-N-684	`-----------------------------------------`
SV1-N-764	`-----------------------------------------`
SV1-N-776	`-----------------------------------------`
Y-797-N1	`-----------------------------------------`
SV1-N-841	`--------------------------------AFIDEAEY-`
SV1-N-845	`-----------------------------------------`

Sequence Logo

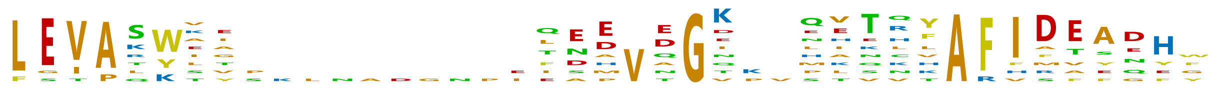

Ruler 2: 1.060 · 1.070 · 1.080

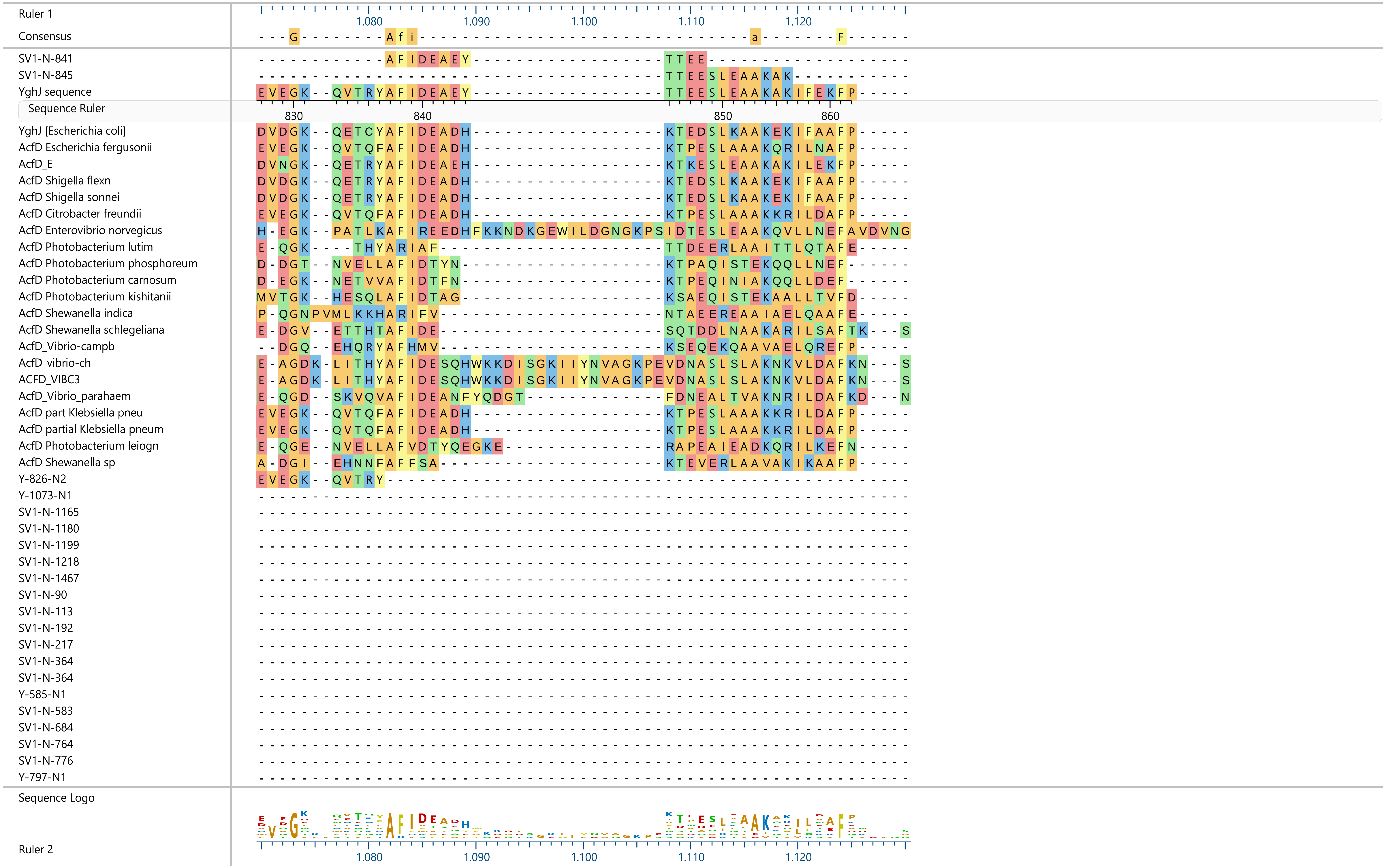

Ruler 1
1.080
1.090
1.100
1.110
1.120
Consensus
Sequence Ruler
830
840
850
860
SV1-N-841
SV1-N-845
YghJ sequence
YghJ [Escherichia coli]
AcfD Escherichia fergusonii
AcfD_E
AcfD Shigella flexn
AcfD Shigella sonnei
AcfD Citrobacter freundii
AcfD Enterovibrio norvegicus
AcfD Photobacterium lutim
AcfD Photobacterium phosphoreum
AcfD Photobacterium carnosum
AcfD Photobacterium kishitanii
AcfD Shewanella indica
AcfD Shewanella schlegeliana
AcfD_Vibrio-campb
AcfD_vibrio-ch_
ACFD_VIBC3
AcfD_Vibrio_parahaem
AcfD part Klebsiella pneu
AcfD partial Klebsiella pneum
AcfD Photobacterium leiogn
AcfD Shewanella sp
Y-826-N2
Y-1073-N1
SV1-N-1165
SV1-N-1180
SV1-N-1199
SV1-N-1218
SV1-N-1467
SV1-N-90
SV1-N-113
SV1-N-192
SV1-N-217
SV1-N-364
SV1-N-364
Y-585-N1
SV1-N-583
SV1-N-684
SV1-N-764
SV1-N-776
Y-797-N1
Sequence Logo
Ruler 2
1.080
1.090
1.100
1.110
1.120

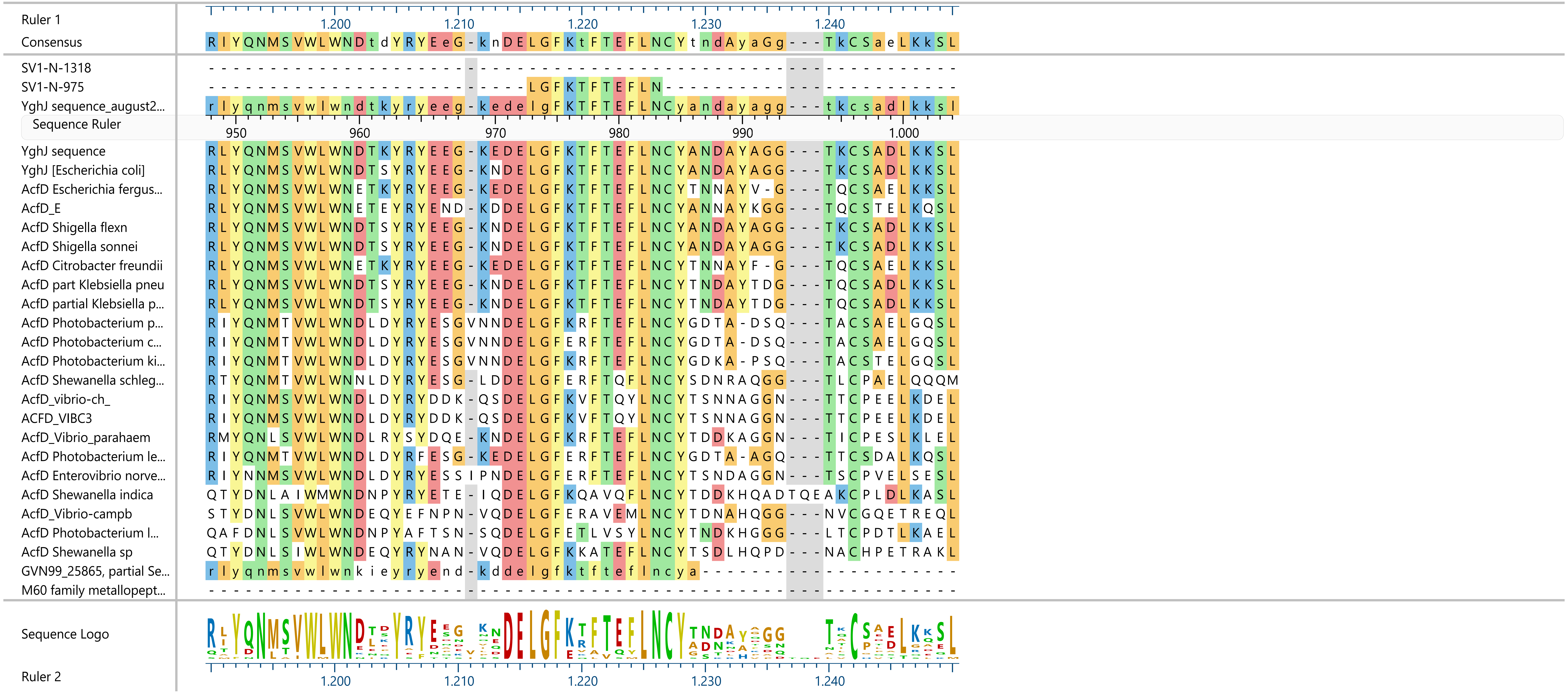

Ruler 1
1.200 1.210 1.220 1.230 1.240
Consensus RlYQNMSVWLWNDtdYRYEeG-knDELGFKtFTEFLNCYtndAyaGg---TkCSaeLKkSL
SV1-N-1318 ---
SV1-N-975 --------------------------LGFKTFTEFLN------------------------
YghJ sequence_august2... rlyqnmsvwlwndtkyryeeg-kedelgFKTFTEFLNCyandayagg---tkcsadlkksl
Sequence Ruler
950 960 970 980 990 1.000
YghJ sequence RLYQNMSVWLWNDTKYRYEEG-KEDELGFKTFTEFLNCYANDAYAGG---TKCSADLKKSL
YghJ [Escherichia coli] RLYQNMSVWLWNDTSYRYEEG-KNDELGFKTFTEFLNCYANDAYAGG---TKCSADLKKSL
AcfD Escherichia fergus... RLYQNMSVWLWNETKYRYEEG-KEDELGFKTFTEFLNCYTNNAYV-G---TQCSAELKKSL
AcfD_E RLYQNMSVWLWNETEYRYEND-KDDELGFKTFTEFLNCYANNAYKGG---TQCSTELKQSL
AcfD Shigella flexn RLYQNMSVWLWNDTSYRYEEG-KNDELGFKTFTEFLNCYANDAYAGG---TKCSADLKKSL
AcfD Shigella sonnei RLYQNMSVWLWNDTSYRYEEG-KNDELGFKTFTEFLNCYANDAYAGG---TKCSADLKKSL
AcfD Citrobacter freundii RLYQNMSVWLWNETKYRYEEG-KEDELGFKTFTEFLNCYTNNAYF-G---TQCSAELKKSL
AcfD part Klebsiella pneu RLYQNMSVWLWNDTSYRYEEG-KNDELGFKTFTEFLNCYTNDAYTDG---TQCSADLKKSL
AcfD partial Klebsiella p... RLYQNMSVWLWNDTSYRYEEG-KNDELGFKTFTEFLNCYTNDAYTDG---TQCSADLKKSL
AcfD Photobacterium p... RIYQNMTVWLWNDLDYRYESGVNNDELGFKRFTEFLNCYGDTA-DSQ---TACSAELGQSL
AcfD Photobacterium c... RIYQNMTVWLWNDLDYRYESGVNNDELGFERFTEFLNCYGDTA-DSQ---TACSAELGQSL
AcfD Photobacterium ki... RIYQNMTVWLWNDLDYRYESGVNNDELGFKRFTEFLNCYGDKA-PSQ---TACSTELGQSL
AcfD Shewanella schleg... RTYQNMTVWLWNNLDYRYESG-LDDELGFERFTQFLNCYSDNRAQGG---TLCPAELQQQM
AcfD_vibrio-ch_ RIYQNMSVWLWNDLDYRYDDK-QSDELGFKVFTQYLNCYTSNNAGGN---TTCPEELKDEL
ACFD_VIBC3 RIYQNMSVWLWNDLDYRYDDK-QSDELGFKVFTQYLNCYTSNNAGGN---TTCPEELKDEL
AcfD_Vibrio_parahaem RMYQNLSVWLWNDLRYSYDQE-KNDELGFKRFTEFLNCYTDDKAGGN---TICPESLKLEL
AcfD Photobacterium le... RIYQNMTVWLWNDLDYRFESG-KEDELGFERFTEFLNCYGDTA-AGQ---TTCSDALKQSL
AcfD Enterovibrio norve... RIYNNMSVWLWNDLDYRYESSIPNDELGFERFTEFLNCYTSNDAGGN---TSCPVELSESL
AcfD Shewanella indica QTYDNLAIWMWNDNPYRYETE-IQDELGFKQAVQFLNCYTDDKHQADTQEAKCPLDLKASL
AcfD_Vibrio-campb STYDNLSVWLWNDEQYEFNPN-VQDELGFERAVEMLNCYTDNAHQGG---NVCGQETREQL
AcfD Photobacterium l... QAFDNLSVWLWNDNPYAFTSN-SQDELGFETLVSYLNCYTNDKHGGG---LTCPDTLKAEL
AcfD Shewanella sp QTYDNLSIWLWNDEQYRYNAN-VQDELGFKKATEFLNCYTSDLHQPD---NACHPETRAKL
GVN99_25865, partial Se... rlyqnmsvwlwnkieyryend-kddelgfktfteflncya---------------------
M60 family metallopept... ---
Sequence Logo
Ruler 2
1.200 1.210 1.220 1.230 1.240

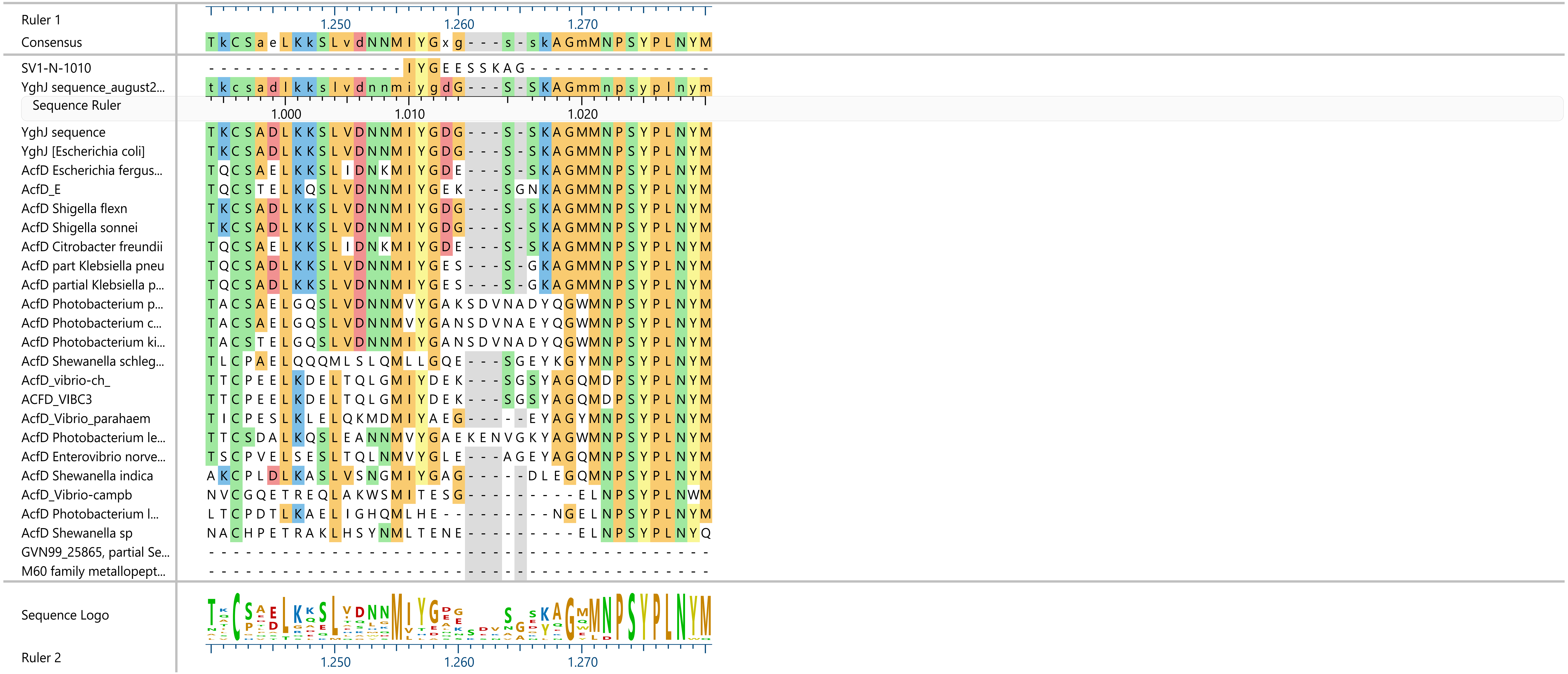

Ruler 1
1.250
1.260
1.270
Consensus
TkCSaeLKkSLvdNNMIYGxg---s-skAGmMNPSYPLNYM
SV1-N-1010
---------------IYGEESSKAG----------------
YghJ sequence_august2...
tkcsadlkkslvdnnmiygdG---S-SKAGmmnpsyplnym
Sequence Ruler
1.000
1.010
1.020
YghJ sequence
TKCSADLKKSLVDNNMIYGDG---S-SKAGMMNPSYPLNYM
YghJ [Escherichia coli]
TKCSADLKKSLVDNNMIYGDG---S-SKAGMMNPSYPLNYM
AcfD Escherichia fergus...
TQCSAELKKSLIDNKMIYGDE---S-SKAGMMNPSYPLNYM
AcfD_E
TQCSTELKQSLVDNNMIYGEK---SGNKAGMMNPSYPLNYM
AcfD Shigella flexn
TKCSADLKKSLVDNNMIYGDG---S-SKAGMMNPSYPLNYM
AcfD Shigella sonnei
TKCSADLKKSLVDNNMIYGDG---S-SKAGMMNPSYPLNYM
AcfD Citrobacter freundii
TQCSAELKKSLIDNKMIYGDE---S-SKAGMMNPSYPLNYM
AcfD part Klebsiella pneu
TQCSADLKKSLVDNNMIYGES---S-GKAGMMNPSYPLNYM
AcfD partial Klebsiella p...
TQCSADLKKSLVDNNMIYGES---S-GKAGMMNPSYPLNYM
AcfD Photobacterium p...
TACSAELGQSLVDNNMVYGAKSDVNADYQGWMNPSYPLNYM
AcfD Photobacterium c...
TACSAELGQSLVDNNMVYGANSDVNAEYQGWMNPSYPLNYM
AcfD Photobacterium ki...
TACSTELGQSLVDNNMIYGANSDVNADYQGWMNPSYPLNYM
AcfD Shewanella schleg...
TLCPAELQQQMLSLQMLLGQE---SGEYKGYMNPSYPLNYM
AcfD_vibrio-ch_
TTCPEELKDELTQLGMIYDEK---SGSYAGQMDPSYPLNYM
ACFD_VIBC3
TTCPEELKDELTQLGMIYDEK---SGSYAGQMDPSYPLNYM
AcfD_Vibrio_parahaem
TICPESLKLELQKMDMIYAEG-----EYAGYMNPSYPLNYM
AcfD Photobacterium le...
TTCSDALKQSLEANNMVYGAEKENVGKYAGWMNPSYPLNYM
AcfD Enterovibrio norve...
TSCPVELSESLTQLNMVYGLE---AGEYAGQMNPSYPLNYM
AcfD Shewanella indica
AKCPLDLKASLVSNGMIYGAG-----DLEGQMNPSYPLNYM
AcfD_Vibrio-campb
NVCGQETREQLAKWSMITESG---------ELNPSYPLNWM
AcfD Photobacterium l...
LTCPDTLKAELIGHQMLHE---------NGELNPSYPLNYM
AcfD Shewanella sp
NACHPETRAKLHSYNMLTENE---------ELNPSYPLNYQ
GVN99_25865, partial Se...

M60 family metallopept...

Sequence Logo
Ruler 2
1.250
1.260
1.270

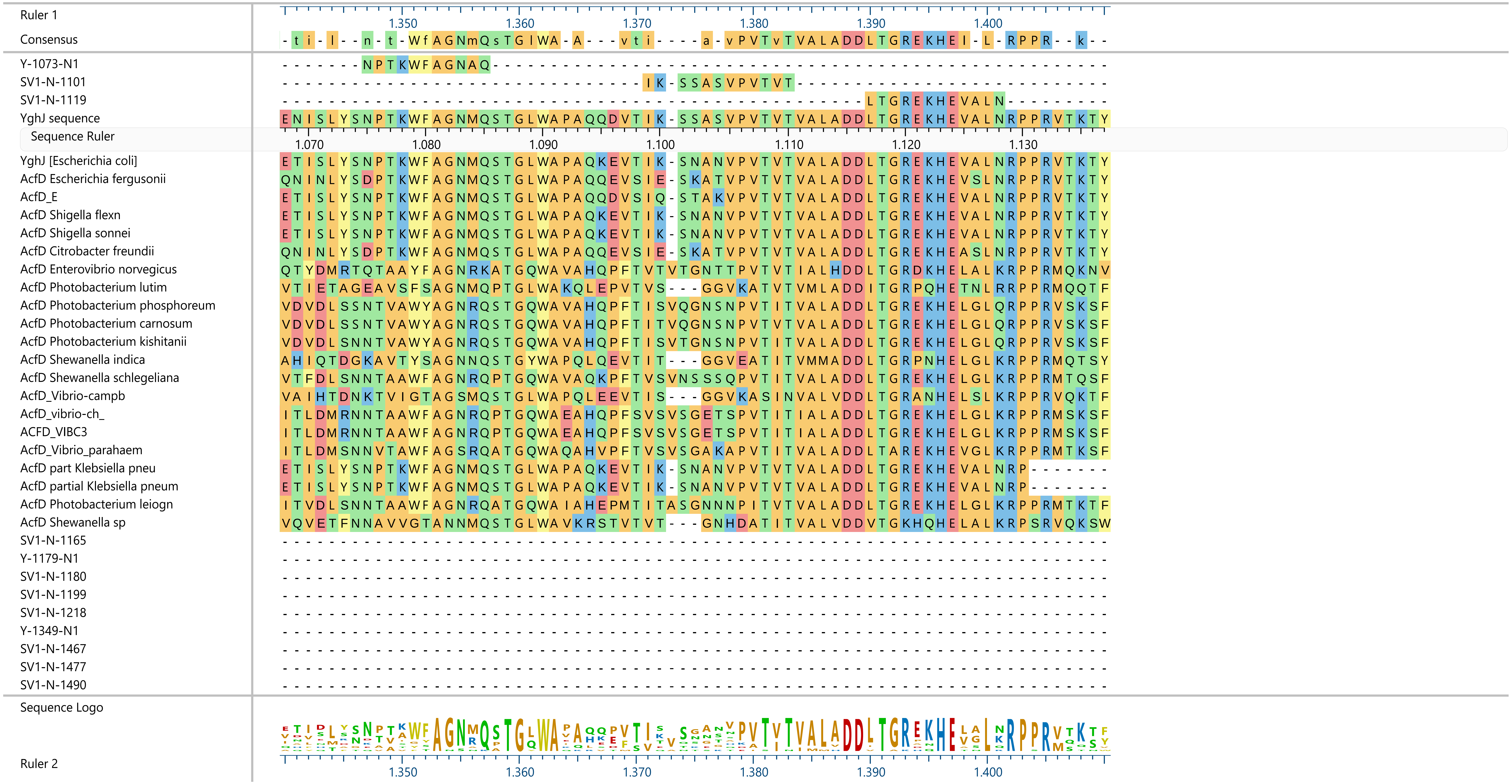
Ruler 1
1.350 1.360 1.370 1.380 1.390 1.400
Consensus - t i - l - - n - t - W f A G N m Q s T G l W A - A - - - v t i - - - - a - v P V T v T V A L A D D L T G R E K H E I - L - R P P R - - k - -
Y-1073-N1 NPTKWFAGNAQ
SV1-N-1101 IK-SSASVPVTVT
SV1-N-1119 LTGREKHEVALN
YghJ sequence ENISLYSNPTKWFAGNMQSTGLWAPAQQDVTIK-SSASVPVTVTVALADDLTGREKHEVALNRPPRVTKTY
Sequence Ruler
1.070 1.080 1.090 1.100 1.110 1.120 1.130
YghJ [Escherichia coli] ETISLYSNPTKWFAGNMQSTGLWAPAQKEVTIK-SNANVPVTVTVALADDLTGREKHEVALNRPPRVTKTY
AcfD Escherichia fergusonii QNINLYSDPTKWFAGNMQSTGLWAPAQQEVSIE-SKATVPVTVTVALADDLTGREKHEVSLNRPPRVTKTY
AcfD_E ETISLYSNPTKWFAGNMQSTGLWAPAQQDVSIQ-STAKVPVTVTVALADDLTGREKHEVALNRPPRVTKTY
AcfD Shigella flexn ETISLYSNPTKWFAGNMQSTGLWAPAQKEVTIK-SNANVPVTVTVALADDLTGREKHEVALNRPPRVTKTY
AcfD Shigella sonnei ETISLYSNPTKWFAGNMQSTGLWAPAQKEVTIK-SNANVPVTVTVALADDLTGREKHEVALNRPPRVTKTY
AcfD Citrobacter freundii QNINLYSDPTKWFAGNMQSTGLWAPAQQEVSIE-SKATVPVTVTVALADDLTGREKHEASLNRPPRVTKTY
AcfD Enterovibrio norvegicus QTYDMRTQTAAYFAGNRKATGQWAVAHQPFTVTVTGNTTPVTVTIALHDDLTGRDKHELALKRPPRMQKNV
AcfD Photobacterium lutim VTIETAGEAVSFSAGNMQPTGLWAKQLEPVTVS---GGVKATVTVMLADDITGRPQHETNLRRPPRMQQTF
AcfD Photobacterium phosphoreum VDVDLSSNTVAWYAGNRQSTGQWAVAHQPFTISVQGNSNPVTITVALADDLTGREKHELGLQRPPRVSKSF
AcfD Photobacterium carnosum VDVDLSSNTVAWYAGNRQSTGQWAVAHQPFTITVQGNSNPVTVTVALADDLTGREKHELGLQRPPRVSKSF
AcfD Photobacterium kishitanii VDVDLSNNTVAWYAGNRQSTGQWAVAHQPFTISVTGNSNPVTITVALADDLTGREKHELGLQRPPRVSKSF
AcfD Shewanella indica AHIQTDGKAVTYSAGNNQSTGYWAPQLQEVTIT---GGVEATITVMMADDLTGRPNHELGLKRPPRMQTSY
AcfD Shewanella schlegeliana VTFDLSNNTAAWFAGNRQPTGQWAVAQKPFTVSVNSSSQPVTITVALADDLTGREKHELGLKRPPRMTQSF
AcfD_Vibrio-campb VAIHTDNKTVIGTAGSMQSTGLWAPQLEEVTIS---GGVKASINVALVDDLTGRANHELSLKRPPRVQKTF
AcfD_vibrio-ch_ ITLDMRNNTAAWFAGNRQPTGQWAEAHQPFSVSVSGETSPVTITIALADDLTGREKHELGLKRPPRMSKSF
ACFD_VIBC3 ITLDMRNNTAAWFAGNRQPTGQWAEAHQPFSVSVSGETSPVTITIALADDLTGREKHELGLKRPPRMSKSF
AcfD_Vibrio_parahaem ITLDMSNNVTAWFAGSRQATGQWAQAHVPFTVSVSGAKAPVTITVALADDLTAREKHEVGLKRPPRMTKSF
AcfD part Klebsiella pneu ETISLYSNPTKWFAGNMQSTGLWAPAQKEVTIK-SNANVPVTVTVALADDLTGREKHEVALNRP-------
AcfD partial Klebsiella pneum ETISLYSNPTKWFAGNMQSTGLWAPAQKEVTIK-SNANVPVTVTVALADDLTGREKHEVALNRP-------
AcfD Photobacterium leiogn ITVDLSNNTAAWFAGNRQATGQWAIAHEPMTITASGNNNPITVTVALADDLTGREKHELGLKRPPRMTKTF
AcfD Shewanella sp VQVETFNNAVVGTANNMQSTGLWAVKRSTVTVT---GNHDATITVALVDDVTGKHQHELALKRPSRVQKSW
SV1-N-1165
Y-1179-N1
SV1-N-1180
SV1-N-1199
SV1-N-1218
Y-1349-N1
SV1-N-1467
SV1-N-1477
SV1-N-1490
Sequence Logo
Ruler 2
1.350 1.360 1.370 1.380 1.390 1.400

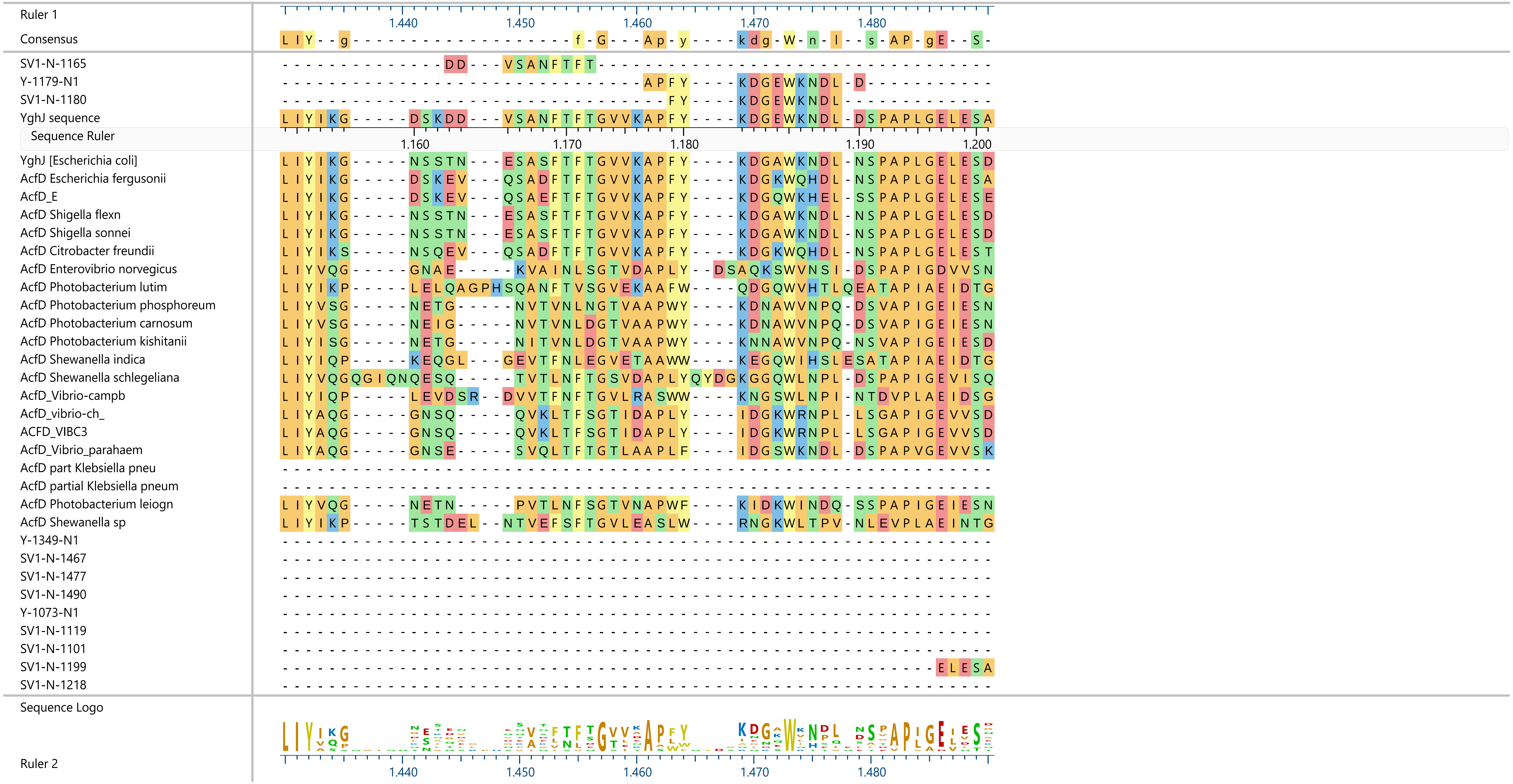
Ruler 1
1.440
1.450
1.460
1.470
1.480
Consensus
SV1-N-1165
Y-1179-N1
SV1-N-1180
YghJ sequence
Sequence Ruler
1.160
1.170
1.180
1.190
1.200
YghJ [Escherichia coli]
AcfD Escherichia fergusonii
AcfD_E
AcfD Shigella flexn
AcfD Shigella sonnei
AcfD Citrobacter freundii
AcfD Enterovibrio norvegicus
AcfD Photobacterium lutim
AcfD Photobacterium phosphoreum
AcfD Photobacterium carnosum
AcfD Photobacterium kishitanii
AcfD Shewanella indica
AcfD Shewanella schlegeliana
AcfD_Vibrio-campb
AcfD_vibrio-ch_
ACFD_VIBC3
AcfD_Vibrio_parahaem
AcfD part Klebsiella pneu
AcfD partial Klebsiella pneum
AcfD Photobacterium leiogn
AcfD Shewanella sp
Y-1349-N1
SV1-N-1467
SV1-N-1477
SV1-N-1490
Y-1073-N1
SV1-N-1119
SV1-N-1101
SV1-N-1199
SV1-N-1218
Sequence Logo
Ruler 2
1.440
1.450
1.460
1.470
1.480

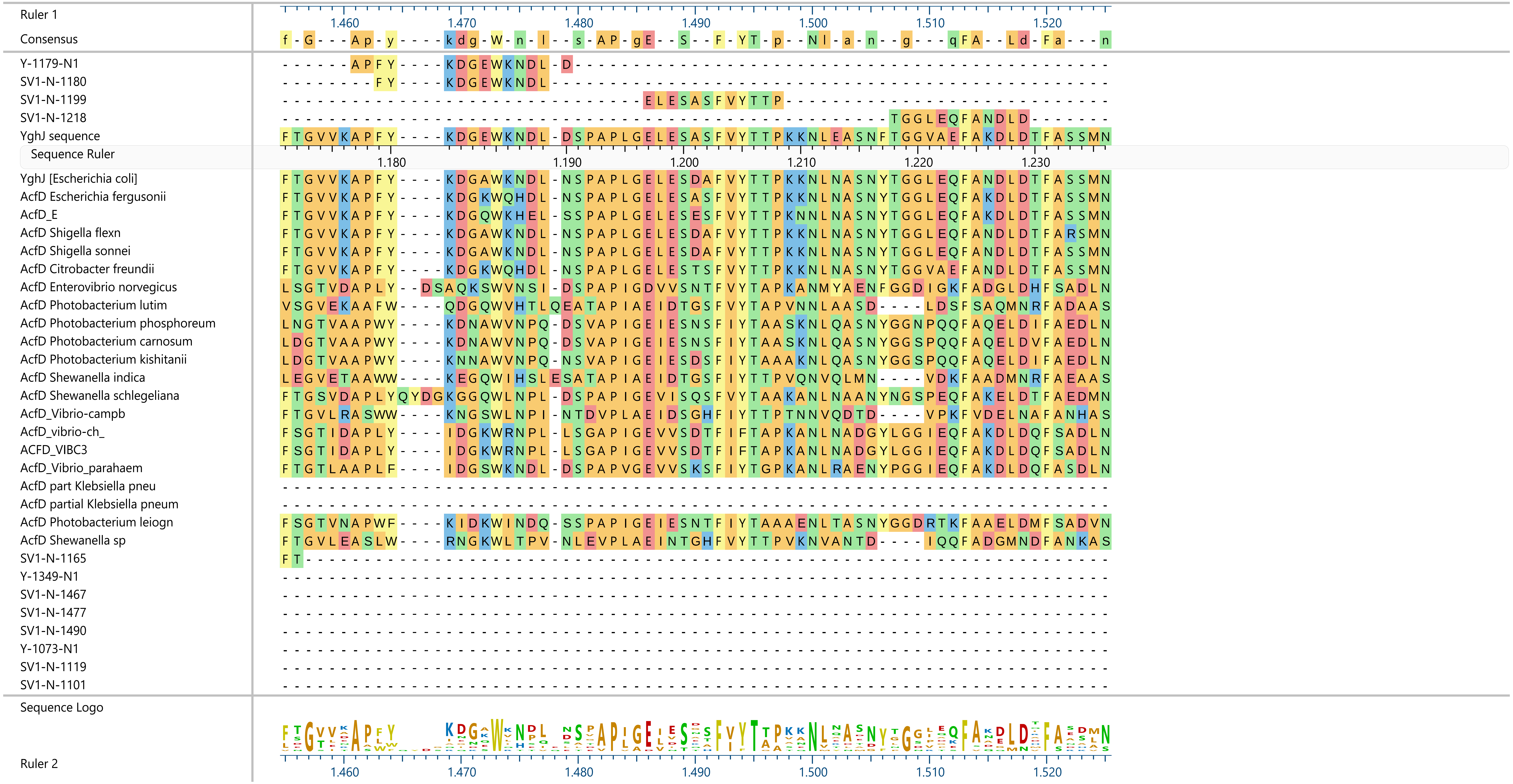

Ruler 1
Consensus
f-G---Ap-y----kdg-W-n-l--s-AP-gE--S--F-YT-p--Nl-a-n--g---qFA--Ld-Fa---n
Y-1179-N1 ------APFY----KDGEWKNDL-D
SV1-N-1180 --------FY----KDGEWKNDL
SV1-N-1199 ELESASFVYTTP
SV1-N-1218 TGGLEQFANDLD
YghJ sequence FTGVVKAPFY----KDGEWKNDL-DSPAPLGELESASFVYTTPKKNLEASNFTGGVAEFAKDLDTFASSMN
Sequence Ruler
YghJ [Escherichia coli] FTGVVKAPFY----KDGAWKNDL-NSPAPLGELESDAFVYTTPKKNLNASNYTGGLEQFANDLDTFASSMN
AcfD Escherichia fergusonii FTGVVKAPFY----KDGKWQHDL-NSPAPLGELESASFVYTTPKKNLNASNYTGGLEQFAKDLDTFASSMN
AcfD_E FTGVVKAPFY----KDGQWKHEL-SSPAPLGELESESFVYTTPKNNLNASNYTGGLEQFAKDLDTFASSMN
AcfD Shigella flexn FTGVVKAPFY----KDGAWKNDL-NSPAPLGELESDAFVYTTPKKNLNASNYTGGLEQFANDLDTFARSMN
AcfD Shigella sonnei FTGVVKAPFY----KDGAWKNDL-NSPAPLGELESDAFVYTTPKKNLNASNYTGGLEQFANDLDTFASSMN
AcfD Citrobacter freundii FTGVVKAPFY----KDGKWQHDL-NSPAPLGELESTSFVYTTPKKNLNASNYTGGVAEFANDLDTFASSMN
AcfD Enterovibrio norvegicus LSGTVDAPLY--DSAQKSWVNSI-DSPAPIGDVVSNTFVYTAPKANMYAENFGGDIGKFADGLDHFSADLN
AcfD Photobacterium lutim VSGVEKAAFW----QDGQWVHTLQEATAPIAEIDTGSFVYTAPVNNLAASD----LDSFSAQMNRFADAAS
AcfD Photobacterium phosphoreum LNGTVAAPWY----KDNAWVNPQ-DSVAPIGEIESNSFIYTAASKNLQASNYGGNPQQFAQELDIFAEDLN
AcfD Photobacterium carnosum LDGTVAAPWY----KDNAWVNPQ-DSVAPIGEIESNSFIYTAASKNLQASNYGGSPQQFAQELDVFAEDLN
AcfD Photobacterium kishitanii LDGTVAAPWY----KNNAWVNPQ-NSVAPIGEIESDSFIYTAAAKNLQASNYGGSPQQFAQELDIFAEDLN
AcfD Shewanella indica LEGVETAAWW----KEGQWIHSLESATAPIAEIDTGSFIYTTPVQNVQLMN----VDKFAADMNRFAEAAS
AcfD Shewanella schlegeliana FTGSVDAPLYQYDGKGGQWLNPL-DSPAPIGEVISQSFVYTAAKANLNAANYNGSPEQFAKELDTFAEDMN
AcfD_Vibrio-campb FTGVLRASWW----KNGSWLNPI-NTDVPLAEIDSGHFIYTTPTNNVQDTD----VPKFVDELNAFANHAS
AcfD_vibrio-ch_ FSGTIDAPLY----IDGKWRNPL-LSGAPIGEVVSDTFIFTAPKANLNADGYLGGIEQFAKDLDQFSADLN
ACFD_VIBC3 FSGTIDAPLY----IDGKWRNPL-LSGAPIGEVVSDTFIFTAPKANLNADGYLGGIEQFAKDLDQFSADLN
AcfD_Vibrio_parahaem FTGTLAAPLF----IDGSWKNDL-DSPAPVGEVVSKSFIYTGPKANLRAENYPGGIEQFAKDLDQFASDLN
AcfD part Klebsiella pneu
AcfD partial Klebsiella pneum
AcfD Photobacterium leiogn FSGTVNAPWF----KIDKWINDQ-SSPAPIGEIESNTFIYTAAAENLTASNYGGDRTKFAAELDMFSADVN
AcfD Shewanella sp FTGVLEASLW----RNGKWLTPV-NLEVPLAEINTGHFVYTTPVKNVANTD----IQQFADGMNDFANKAS
SV1-N-1165 FT
Y-1349-N1
SV1-N-1467
SV1-N-1477
SV1-N-1490
Y-1073-N1
SV1-N-1119
SV1-N-1101
Sequence Logo
Ruler 2
1.460 1.470 1.480 1.490 1.500 1.510 1.520

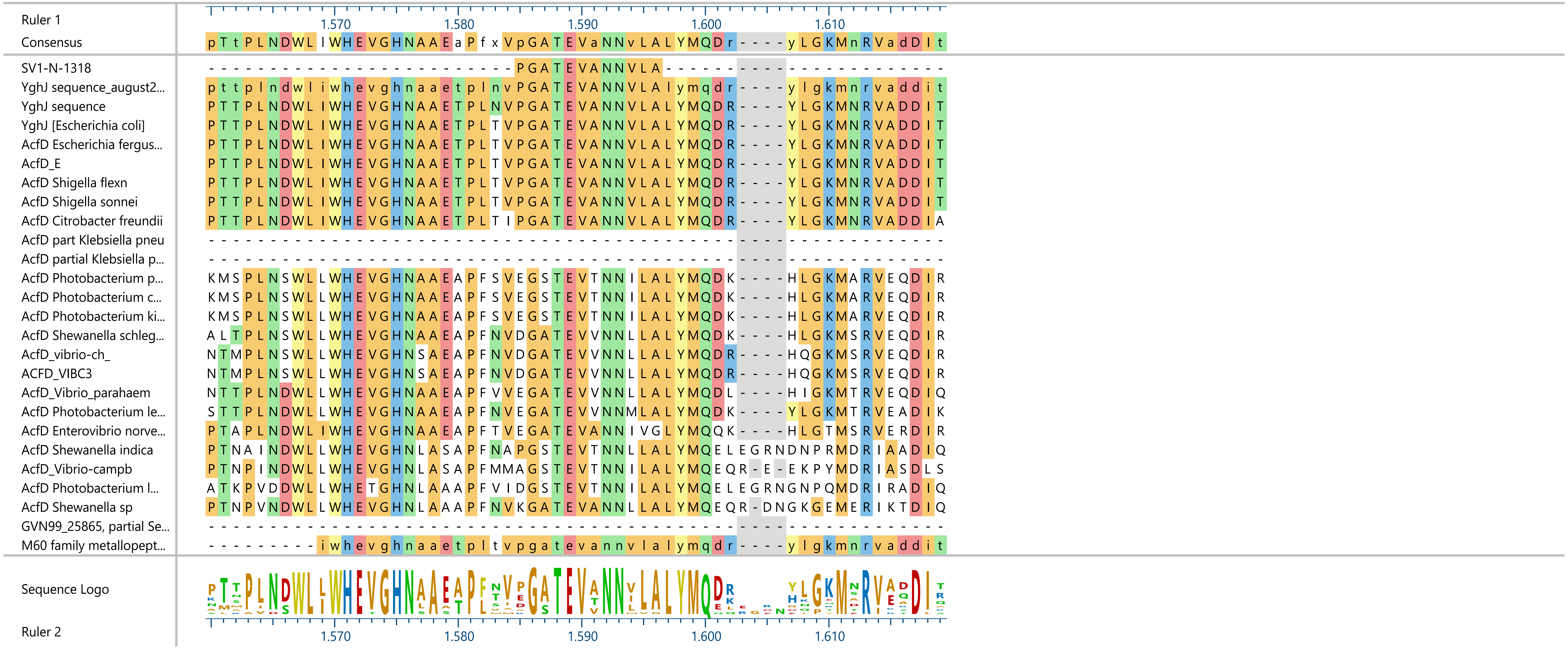
Ruler 1
Consensus
1.570
1.580
1.590
1.600
1.610
SV1-N-1318
YghJ sequence_august2...
YghJ sequence
YghJ [Escherichia coli]
AcfD Escherichia fergus...
AcfD_E
AcfD Shigella flexn
AcfD Shigella sonnei
AcfD Citrobacter freundii
AcfD part Klebsiella pneu
AcfD partial Klebsiella p...
AcfD Photobacterium p...
AcfD Photobacterium c...
AcfD Photobacterium ki...
AcfD Shewanella schleg...
AcfD_vibrio-ch_
ACFD_VIBC3
AcfD_Vibrio_parahaem
AcfD Photobacterium le...
AcfD Enterovibrio norve...
AcfD Shewanella indica
AcfD_Vibrio-campb
AcfD Photobacterium l...
AcfD Shewanella sp
GVN99_25865, partial Se...
M60 family metallopept...
Sequence Logo
Ruler 2

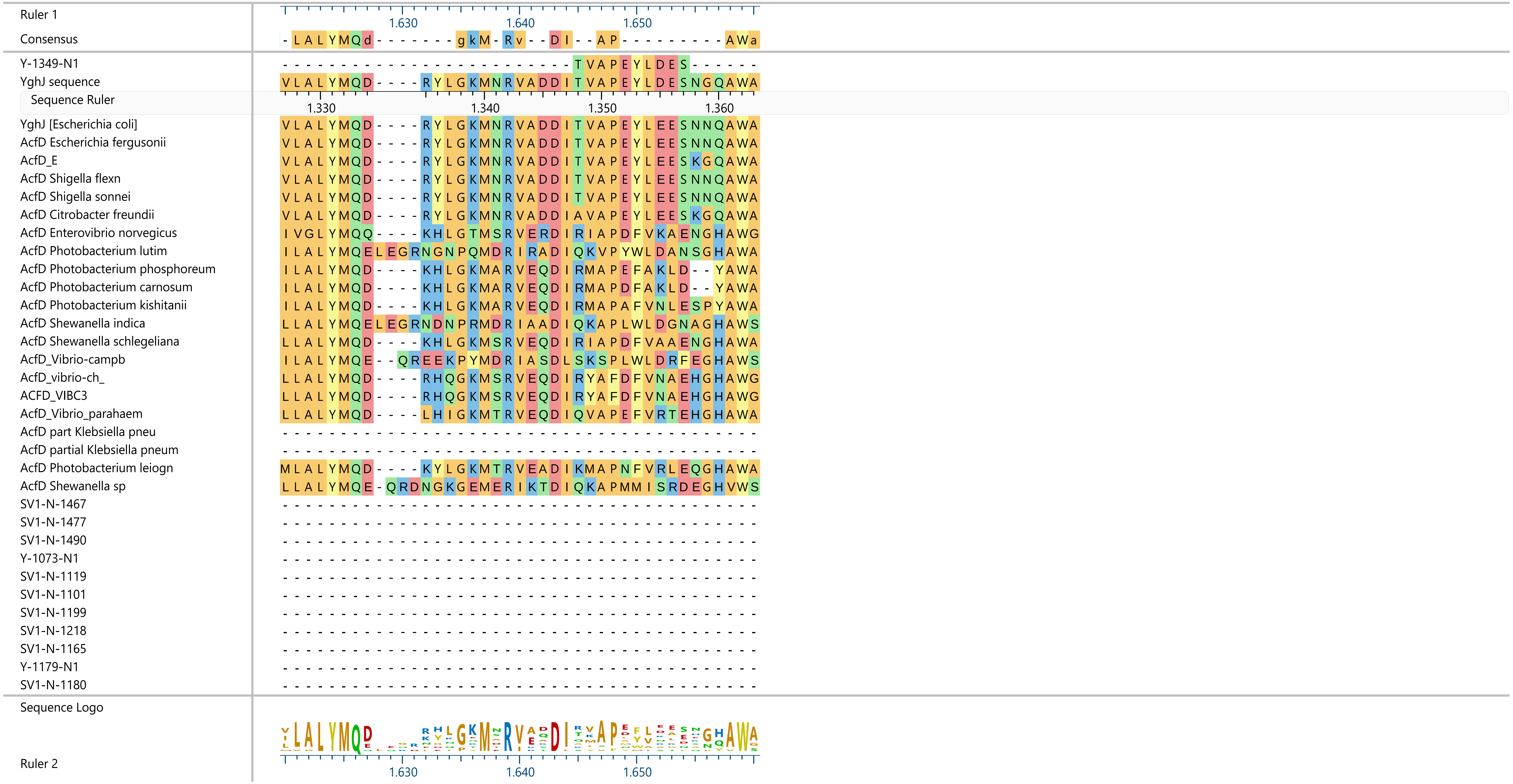

Ruler 1
1.630
1.640
1.650
Consensus -LALYMQd-------gkM-Rv--DI--AP----------AWa
Y-1349-N1 -------------------------TVAPEYLDES------
YghJ sequence VLALYMQD----RYLGKMNRVADDITVAPEYLDESNGQAWA
Sequence Ruler
1.330
1.340
1.350
1.360
YghJ [Escherichia coli] VLALYMQD----RYLGKMNRVADDITVAPEYLEESNNQAWA
AcfD Escherichia fergusonii VLALYMQD----RYLGKMNRVADDITVAPEYLEESNNQAWA
AcfD_E VLALYMQD----RYLGKMNRVADDITVAPEYLEESKGQAWA
AcfD Shigella flexn VLALYMQD----RYLGKMNRVADDITVAPEYLEESNNQAWA
AcfD Shigella sonnei VLALYMQD----RYLGKMNRVADDITVAPEYLEESNNQAWA
AcfD Citrobacter freundii VLALYMQD----RYLGKMNRVADDIAVAPEYLEESKGQAWA
AcfD Enterovibrio norvegicus IVGLYMQQ----KHLGTMSRVERDIRIAPDFVKAENGHAWG
AcfD Photobacterium lutim ILALYMQELEGRNGNPQMDRIRADIQKVPYWLDANSGHAWA
AcfD Photobacterium phosphoreum ILALYMQD----KHLGKMARVEQDIRMAPEFAKLD--YAWA
AcfD Photobacterium carnosum ILALYMQD----KHLGKMARVEQDIRMAPDFAKLD--YAWA
AcfD Photobacterium kishitanii ILALYMQD----KHLGKMARVEQDIRMAPAFVNLESPYAWA
AcfD Shewanella indica LLALYMQELEGRNDNPRMDRIAADIQKAPLWLDGNAGHAWS
AcfD Shewanella schlegeliana LLALYMQD----KHLGKMSRVEQDIRIAPDFVAAENGHAWA
AcfD_Vibrio-campb ILALYMQE--QREEKPYMDRIASDLSKSPLWLDRFEGHAWS
AcfD_vibrio-ch_ LLALYMQD----RHQGKMSRVEQDIRYAFDFVNAEHGHAWG
ACFD_VIBC3 LLALYMQD----RHQGKMSRVEQDIRYAFDFVNAEHGHAWG
AcfD_Vibrio_parahaem LLALYMQD----LHIGKMTRVEQDIQVAPEFVRTEHGHAWA
AcfD part Klebsiella pneu ---
AcfD partial Klebsiella pneum ---
AcfD Photobacterium leiogn MLALYMQD----KYLGKMTRVEADIKMAPNFVRLEQGHAWA
AcfD Shewanella sp LLALYMQE-QRDNGKGEMERIKTDIQKAPMMISRDEGHVWS
SV1-N-1467 ---
SV1-N-1477 ---
SV1-N-1490 ---
Y-1073-N1 ---
SV1-N-1119 ---
SV1-N-1101 ---
SV1-N-1199 ---
SV1-N-1218 ---
SV1-N-1165 ---
Y-1179-N1 ---
SV1-N-1180 ---
Sequence Logo
Ruler 2
1.630
1.640
1.650

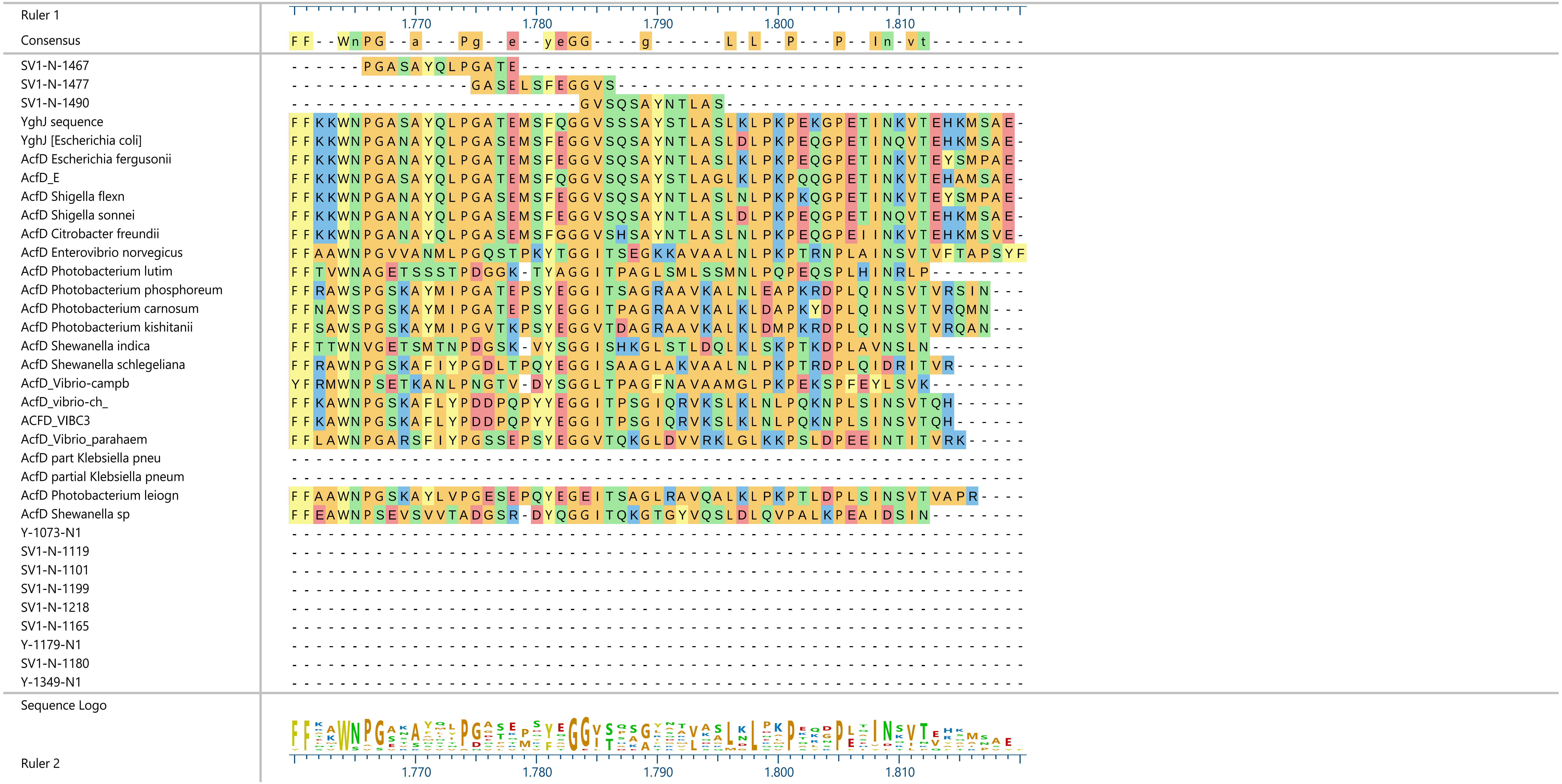

Ruler 1
1.770
1.780
1.790
1.800
1.810
Consensus
SV1-N-1467
SV1-N-1477
SV1-N-1490
YghJ sequence
YghJ [Escherichia coli]
AcfD Escherichia fergusonii
AcfD_E
AcfD Shigella flexn
AcfD Shigella sonnei
AcfD Citrobacter freundii
AcfD Enterovibrio norvegicus
AcfD Photobacterium lutim
AcfD Photobacterium phosphoreum
AcfD Photobacterium carnosum
AcfD Photobacterium kishitanii
AcfD Shewanella indica
AcfD Shewanella schlegeliana
AcfD_Vibrio-campb
AcfD_vibrio-ch_
ACFD_VIBC3
AcfD_Vibrio_parahaem
AcfD part Klebsiella pneu
AcfD partial Klebsiella pneum
AcfD Photobacterium leiogn
AcfD Shewanella sp
Y-1073-N1
SV1-N-1119
SV1-N-1101
SV1-N-1199
SV1-N-1218
SV1-N-1165
Y-1179-N1
SV1-N-1180
Y-1349-N1
Sequence Logo
Ruler 2
